# The Complex Path from Mammary Ductal Hyperplasia to Breast Cancer: Elevated Malignancy Risk in Atypical Forms

**DOI:** 10.3390/biomedicines14020349

**Published:** 2026-02-02

**Authors:** Bogdan-Alexandru Gheban, Lavinia Patricia Mocan, Adina Bianca Boșca, Rada Teodora Suflețel, Eleonora Dronca, Mihaela Elena Jianu, Carmen Crivii, Tudor Cristian Pașcalău, Mădălin Mihai Onofrei, Andreea Moise-Crintea, Alina Simona Șovrea

**Affiliations:** 1 Discipline of Fundamental Sciences, Faculty of Medical Assistance and Health Sciences, Iuliu Hațieganu University of Medicine and Pharmacy, Emil Isaac nr. 8, 400023 Cluj-Napoca, Romania; gheban.bogdan@umfcluj.ro; 2 Department of Pathology, County Emergency Clinical Hospital, Clinicilor 3-5, 400347 Cluj-Napoca, Romania; 3 Discipline of Histology, Faculty of Medicine, Iuliu Hațieganu University of Medicine and Pharmacy, Pasteur nr. 6, 400023 Cluj-Napoca, Romania; bianca.bosca@umfcluj.ro (A.B.B.); sufletel_rada@yahoo.com (R.T.S.); jianu.mihaela21@gmail.com (M.E.J.); tudor.pascalau@umfcluj.ro (T.C.P.); madalin.onofrei@umfcluj.ro (M.M.O.); alina.simona.sovrea01@gmail.com (A.S.Ș.); 4 Discipline of Genetics, Faculty of Medicine, Iuliu Hațieganu University of Medicine and Pharmacy, Pasteur nr. 6, 400023 Cluj-Napoca, Romania; nora_dronca@yahoo.com; 5 Discipline of Anatomy, Faculty of Medicine, Iuliu Hațieganu University of Medicine and Pharmacy, Clinicilor nr. 3-5, 400023 Cluj-Napoca, Romania; carmen.crivii@gmail.com; 6 Discipline of Medical Biochemistry, Faculty of Medicine, Iuliu Hațieganu University of Medicine and Pharmacy, Pasteur nr. 6, 400023 Cluj-Napoca, Romania; crinteaandreea@gmail.com

**Keywords:** mammary ductal hyperplasia, malignant transformation, genetic alterations, breast cancer, diet, environmental factors

## Abstract

Background: Mammary ductal hyperplasia represents a spectrum of benign proliferative breast lesions, some of which pose elevated risks for malignant transformation into ductal carcinoma in situ and invasive breast cancer. This narrative review explores why only specific types, particularly those with atypia, exhibit higher progression potential, synthesizing epidemiologic, histopathologic, molecular, and environmental insights. Methods: We reviewed key literature from databases, including PubMed, focusing on classification, risk stratification, genetic/epigenetic mechanisms, tumor microenvironment dynamics, and modifiable factors influencing progression. Results: Benign breast lesions are categorized into non-proliferative, proliferative without atypia, and proliferative with atypia, such as atypical ductal hyperplasia and atypical lobular hyperplasia. Atypia represents a morphologic continuum toward low-grade ductal carcinoma in situ, driven by genetic alterations, epigenetic reprogramming, and changes in the tumor microenvironment, including stromal remodeling, immune infiltration, hypoxia-induced angiogenesis, and extracellular matrix degradation. Dietary factors, such as high-fat intake and obesity, exacerbate progression through inflammation, insulin resistance, and adipokine imbalance, while environmental toxins, including endocrine disruptors, pesticides, and ionizing radiation, amplify genomic instability. Conclusions: Understanding differential risks and mechanisms underscores the need for stratified surveillance, biomarker-driven interventions, and lifestyle modifications to mitigate progression. Future research should prioritize molecular profiling for personalized prevention in high-risk hyperplasia.

## 1. Introduction

Mammary ductal hyperplasia (MDH) is a condition characterized by the abnormal growth of cells within the breast’s milk ducts. This condition can vary in severity, and while it is considered a benign lesion, it is often regarded as a potential precursor or risk factor for the development of breast carcinoma, primarily when associated with other pathological changes. The relationship between different types of mammary hyperplasia and the subsequent development of carcinoma is complex, influenced by both genetic and environmental factors.

Recent studies have further elucidated the progression risks associated with MDH, particularly emphasizing the role of atypical ductal hyperplasia (ADH) as a high-risk lesion. A 2023 study highlights that ADH confers a 4- to 5-fold increased risk of developing ductal carcinoma in situ (DCIS) within five years, positioning it as a marker of elevated breast cancer susceptibility rather than a direct precursor, with the risk manifesting bilaterally in the breasts [1]. A 2020 analysis indicated that the breast cancer development rate during surveillance after ADH surgery was 7.3% over a mean follow-up of 63.9 months, with palpable lesions showing significantly higher rates of progression [2]. A 2020 focused review on B3 lesions with atypia supports a low-grade progression model for ADH, noting the need for careful risk assessment to avoid overdiagnosis [3]. A 2021 review on high-risk breast lesions advocates for multidisciplinary management, including risk assessment tools and chemoprevention, to mitigate progression in atypical hyperplasia (AH) cases [4].

This narrative review synthesizes epidemiologic, histopathologic, molecular, and environmental insights to explore why only specific types of hyperplasia, particularly those with atypia, exhibit higher malignant potential. By delineating the mechanisms underlying differential progression risks, we aim to inform stratified surveillance strategies, biomarker-driven interventions, and lifestyle modifications for high-risk patients.

## 2. Materials and Methods

This narrative review was conducted to synthesize current evidence on the progression of MDH to breast cancer, with a focus on differential risks among hyperplasia subtypes and underlying mechanisms. The review adheres to general guidelines for narrative reviews in medical literature, emphasizing transparency in the literature selection and synthesis without formal meta-analysis or systematic appraisal tools such as PRISMA.

### 2.1. Literature Search Strategy

A comprehensive literature search was performed between January 2025 and November 2025. PubMed/MEDLINE served as the primary database, supplemented by manual searches of reference lists from key articles for additional relevant publications. The search strategy employed a combination of Medical Subject Headings. Key search terms included: (“ductal hyperplasia” OR “atypical ductal hyperplasia” OR “atypical hyperplasia” OR “flat epithelial atypia” OR “usual ductal hyperplasia” OR “proliferative breast disease”) AND (“breast cancer risk” OR “malignant progression” OR “precursor lesion” OR “genetic alteration” OR “PIK3CA” OR “tumor microenvironment” OR “stromal remodeling” OR “immune infiltration” OR “angiogenesis” OR “diet” OR “obesity” OR “endocrine disruptors” OR “environmental factors”). No date restrictions were imposed on the electronic search to capture foundational studies; however, priority was given to articles published after 2020 to emphasize recent advancements in molecular and environmental insights.

### 2.2. Inclusion and Exclusion Criteria

Studies were included if they were peer-reviewed articles published in English and provided relevant data on the histologic classification, risk stratification, genetic/epigenetic mechanisms, tumor microenvironment dynamics, dietary/obesity influences, or environmental factors related to the progression from mammary hyperplasia to carcinoma. Exclusion criteria encompassed conference abstracts, case reports, editorials, letters, non-English publications, and studies lacking direct relevance to hyperplasia progression.

### 2.3. Study Selection and Data Synthesis

Initial search results yielded over 220 references, which were screened for relevance by title and abstract. Full-text articles were retrieved for detailed evaluation, resulting in 209 citations ultimately included. Data were synthesized narratively, grouping findings thematically (e.g., classification, genetic drivers, microenvironment, and modifiable factors) to highlight mechanistic coherence and translational potential. Figures illustrating key concepts were generated using BioRender software under a publishing license CC BY 4.0.

## 3. Mammary Ductal Hyperplasia (MDH) and Its Types

Breast carcinogenesis is often framed as a multistep evolution from benign epithelial alterations toward DCIS and eventually invasive breast carcinoma. Yet in practice, most benign epithelial proliferations remain morphologically stable throughout a woman’s lifetime. Discriminating between proliferative lesions that are genuinely at risk and those that are innocuous is fundamental to guiding surveillance, biopsy decisions, and chemoprevention strategies. A proper conceptual schema subdivides benign mammary epithelial alterations into three broad categories:

- Nonproliferative changes;
- Proliferative hyperplasia without cytologic atypia;
- Proliferative hyperplasia with cytologic atypia [5].

In what follows, we review key entities in each category, summarize the epidemiologic evidence on differential cancer risk, and explore plausible mechanistic explanations for why only specific hyperplastic lesions tend to evolve.

### 3.1. Classification of Benign Breast Lesions and Associated Risks

#### 3.1.1. Nonproliferative Changes

Nonproliferative lesions are those lacking significant epithelial proliferation. Representative lesions include simple cysts, ductal ectasia, apocrine metaplasia, mild epithelial hyperplasia without proliferation, and stromal fibrosis [6]. In large cohorts, women whose benign biopsy reveals only nonproliferative changes do not display a significantly increased risk of subsequent breast cancer relative to the background population. Indeed, nonproliferative disease is used as the baseline reference in risk stratification in many studies [7]. In select analyses, marginal associations between specific nonproliferative features (e.g., microcalcifications, mild epithelial change) and later cancer risk have been reported. However, effect sizes are modest and often attenuate with multivariable adjustment. In the classic Hartmann et al. cohort of 9087 women with benign breast disease, nonproliferative lesions had a relative risk (RR) for breast cancer of 1.27 (95% CI 1.15–1.41) compared to the general population, which is lower than in proliferative disease without atypia (RR ~ 1.88) or AH (RR ~ 4.24) [3].

A recent systematic review emphasized that the breast cancer risk associated with benign breast disease is heterogeneous, primarily determined by the extent of epithelial proliferation and the presence or absence of cytologic atypia. While benign breast disease in general correlates with an elevated incidence of breast cancer compared to women without such findings, the magnitude of this increase is substantially lower for nonproliferative lesions than for either proliferative lesions without atypia or AH [8]. Consequently, nonproliferative disease represents the lowest-risk category within the benign breast disease spectrum.

Thus, the consensus is that nonproliferative lesions are low-risk and do not demand intensive follow-up beyond standard screening, unless modified by other risk factors (e.g., family history, dense breasts, reproductive and hormonal factors, or genetic predisposition) [9,10].

#### 3.1.2. Proliferative Hyperplasia Without Atypia

This category encompasses lesions with increased epithelial cell numbers but without definite cytologic atypia. Key entities include: usual ductal hyperplasia (UDH), sclerosing adenosis, radial scars, complex sclerosing lesions, intraductal papillomas without atypia, complex proliferative lesions, or multifocal proliferative foci.

Epidemiologic evidence indicates that proliferative lesions without atypia confer a modest increase in breast cancer risk, typically in the range of 1.5- to 2.0-fold compared to nonproliferative disease. For example, in a large case–control study nested within benign breast disease cohorts, proliferative lesions without atypia had an adjusted odds ratio (OR) of approximately 1.45 relative to nonproliferative disease. [11]. Other classic studies similarly estimate a RR of 1.8–1.9 [3].

With a focus solely on certain lesional entities, UDH is characterized by a haphazard proliferation of heterogeneous epithelial cells forming slit-like spaces, bridging projections, and irregular architecture. Because of its disorganized morphology and lack of monoclonal dominance, UDH is generally considered a marker of increased risk rather than a direct precursor lesion. [3,12]. Currently, no reliable prognostic indicators exist to determine which patients with UDH will progress to invasive breast carcinoma. The associated risk level is comparable to that conferred by specific reproductive characteristics, such as early onset of menarche or delayed menopause. It therefore does not justify the modification of routine mammographic screening intervals [13].

Radial scars and complex sclerosing lesions often harbor adjacent proliferative changes, and some series report modest independent risk contributions or occasional occult carcinoma [14]. Although once considered potential precursors, current evidence does not confirm a premalignant nature for radial scars. Their modestly increased breast cancer risk (RR of approximately 1.4–1.7) likely reflects coexisting proliferative changes rather than intrinsic malignancy. Larger lesions (>0.6 cm), more common in women over 50, may coexist with AH or carcinoma [15]. Optimal management of mammographically detected radial scars and complex sclerosing lesions remains debated. Lesions showing epithelial atypia on core biopsy carry a malignancy risk warranting excision [16,17]. In contrast, recent studies suggest that lesions with concordant imaging and pathologic aspects lacking atypia might be managed conservatively [18,19,20].

To sum up, proliferative disease without atypia constitutes an intermediate-risk stratum but, by itself, lacks many of the hallmarks of progression to carcinoma, such as architectural disorder or clonal expansion.

#### 3.1.3. Proliferative Hyperplasia with Atypia

This is the high-risk category, containing principally ADH and atypical lobular hyperplasia (ALH), and, in some classification schemes, flat epithelial atypia (FEA). These lesions combine proliferation with cytologic uniformity, partial loss of polarity, and architectural disturbance.

AH is identified in 4 to 10% of benign breast biopsies performed for imaging or palpable lesions [5,9]. Multiple long-term follow-up cohorts and meta-analyses demonstrate that proliferative hyperplasia with atypia confers a 3- to 5-fold RR of future breast carcinoma compared to nonproliferative disease [3,21,22,23]. In the Mayo cohort, the estimated cumulative incidence of breast cancer at 25 years in women with AH was approximately 29% [9]. Recent large-scale analyses using contemporary imaging and biopsy data refined these estimates. In the Breast Cancer Surveillance Consortium cohort, the 10-year incidence of invasive carcinoma after ADH was approximately 5 to 6%, slightly lower than previous figures, with risk modestly higher in excisional than in core needle biopsies (6.7% vs. 5%) [24]. These findings suggest that smaller ADH foci, increasingly detected by modern imaging, may carry a lower absolute risk than in earlier cohorts. However, ADH remains a significant marker of long-term susceptibility [25].

ALH is the lobular counterpart of ADH, arising in the terminal duct-lobular units. It is characterized by a monomorphic proliferation of discohesive, dyshesive cells (often due to loss of E-cadherin expression) filling part of the lobule. ALH and the related LCIS are collectively referred to as lobular neoplasia, which confers a risk of subsequent carcinoma similar to that of ADH [25].

The often-quoted approximately 1% annual risk for AH is a useful heuristic, though actual risk is modulated by age, family history, breast density, and other lesion features [25].

#### 3.1.4. Subtype Nuances: FEA, ADH vs. ALH, and the Extent of Atypia

FEA represents clonal proliferation of mildly atypical epithelial cells lining terminal duct lobular units, typically associated with microcalcifications and incidentally discovered during mammographic screening [26]. Although limited data exist, evidence indicates that FEA may occasionally precede invasive breast carcinoma [27]. Nonetheless, the probability of progression is very low, and its risk magnitude is considerably lower than that of ADH or ALH [28]. Consequently, FEA should not be managed identically to these higher-risk atypical proliferations.

Several retrospective analyses have reported that up to one-third of FEA lesions identified on core needle biopsy may be more advanced at the time of surgical excision. However, these findings are inconsistent due to small sample sizes, variable histologic criteria, and differences in radiologic–pathologic correlation, resulting in wide variability in reported upgrade rates. Current evidence suggests that surgical excision is not uniformly required after a diagnosis of pure FEA, particularly when post-biopsy imaging confirms complete removal of the calcifications associated with the lesion. Instead, careful radiologic–pathologic concordance is advised to determine appropriate follow-up and to ensure that management remains individualized and evidence-based [29,30].

ADH is frequently conceptualized as a direct, though non-obligate precursor of low-grade DCIS, whereas ALH is considered a risk indicator or a marker of a susceptible field [31]. In practice, both types are associated with subsequent ductal carcinoma, and their clinical outcomes are often similar [27].

The question of whether the extent (number of foci) of atypia modulates risk has been explored. In the nested case–control study by Collins et al., among women with ADH, increasing the number of foci (≥3) did not significantly increase OR beyond that of 1–2 foci (OR 2.7 vs. 3.5, *p* = 0.58) [32]. In ALH, there was a trend toward higher OR with ≥3 foci (OR 8.0) vs. 1–2 foci (OR 5.2), though this was not statistically robust (*p* = 0.66). Other series suggest some stratification by extent, but the evidence is not consistent enough to uniformly guide management [33]. In another systematic review of determinants of breast cancer risk in women with atypia, use of menopausal hormone therapy, timing of follow-up, and other factors were also modifiers of risk [8].

#### 3.1.5. Upgrade Rates and Interpretive Caution

A recurring complication is that many cases of AH diagnoses on core-needle biopsy are upgraded to DCIS or invasive carcinoma on subsequent excisional pathology. This upgrade phenomenon clouds the boundary between actual progression versus initial under-sampling of coexistent malignancy [21]. Meta-analyses of core-biopsy-diagnosed ADH yield upgrade rates to malignancy in the range of 10–30%, depending on biopsy technique, lesion characteristics, and institutional practice [34]. Disentangling upgrades from genuine evolution remains an ongoing challenge, requiring rigorous pathological and imaging correlation and thoughtful clinical decision-making.

Therefore, the histologic classification of benign breast lesions remains highly relevant to stratified surveillance. For nonproliferative disease, standard screening per population guidelines is generally appropriate. In proliferative diseases without atypia, a modest risk elevation may justify closer surveillance in the context of additional risk factors. On the other hand, because AH confers substantial long-term risk, many centers recommend enhanced surveillance (annual mammography plus supplemental MRI) and consideration of risk-reducing interventions. Excision of the atypical lesion does not eliminate the residual elevated risk conferred by the breast environment; therefore, continued vigilance is required [22,35].

### 3.2. Mechanistic Frameworks Underlying the Progression of Breast Lesions

**Figure 1:**
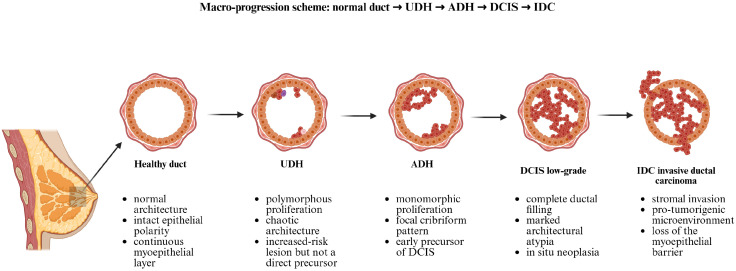
Progression of ductal hyperplasia. Created in BioRender. Elena Mihaela, J. (2025) https://BioRender.com/innlvj8, accessed on 26 November 2025. Figure legend: UDH—usual ductal hyperplasia; ADH—atypical ductal hyperplasia; DCIS—ductal carcinoma in situ; IDC—invasive ductal carcinoma.

Although the above classification and epidemiologic data stratify risk, they do not explain why only particular lesions (especially those with atypia) tend to evolve toward malignancy. Below, we synthesize current mechanistic hypotheses, enriched by recent molecular studies, and present them graphically in Figure 1.

#### 3.2.1. Degree of Atypia

The presence of cytologic and architectural atypia is perhaps the single most important histologic feature distinguishing benign proliferations that merely signify elevated risk from those that embody early steps in the neoplastic continuum. Within AH, the severity of cytologic atypia may correlate with proximity to malignant features (e.g., higher nuclear grade, more mitotic activity, loss of polarity). Lesions with more pronounced atypia may be closer to the threshold and thus more likely to cross into carcinoma, especially with additional alterations [36].

#### 3.2.2. Morphologic and Histopathologic Continuum: ADH as a “Mini-DCIS”

ADH is generally regarded as a transitional lesion within the biological spectrum of breast epithelial proliferations, representing an intermediate step between UDH and low-grade DCIS. One of the most compelling arguments that atypia represents a transitional state is the striking morphologic and structural affinity between ADH and low-grade DCIS. In fact, many pathologists conceptually regard ADH as a limited, partial, or incipient DCIS, fulfilling many of the same architectural and cytologic criteria, but lacking the extent or completeness of ductal involvement [37].

Microscopically, ADH is characterized by a relatively uniform (monomorphic) epithelial population showing mild to moderate nuclear enlargement and minimal pleomorphism. The architectural arrangement often mirrors that of low-grade DCIS, displaying cribriform, micropapillary, or solid patterns; however, these features are typically focal, involving only a limited number of ductal spaces. A partial loss of polarity and reorganization of epithelial layers may also be observed, features that distinguish ADH from the irregular, haphazard cell stratification seen in UDH. From an immunophenotypic standpoint, ADH shares several markers with low-grade DCIS [38]. The atypical cells commonly express estrogen and progesterone receptors and demonstrate a relatively low proliferative index (Ki-67), though this is higher than that observed in non-atypical proliferations. Molecular studies further reveal overlapping genetic and epigenetic alterations between ADH and DCIS, reinforcing the notion that ADH represents an early, quantitatively limited precursor in the neoplastic progression toward DCIS [39].

Because ADH clones possess many (though not all) features of low-grade DCIS, they are anatomically and biologically poised for further progression. Indeed, the threshold from ADH to low-grade DCIS is sometimes subtle and somewhat arbitrary (extent, degree of involvement, continuity) in diagnostic practice [21,40]. Thus, from a morphologic standpoint, the presence of atypia pushes a lesion from the benign proliferative region toward the in situ neoplastic region on the histologic continuum.

#### 3.2.3. Epidemiologic and Clinical Evidence: Progression Risk Linked to Atypia

From the population and cohort studies, the differential risk conferred by AH (vs. proliferative disease without atypia) is pronounced and consistent. Whereas non-atypical proliferative disease confers a RR of approximately 1.5 to 2, AH confers approximately 4–5× risk of future carcinoma [9]. Moreover, long-term follow-up data support that AH is a strong independent predictor of later DCIS and invasive carcinoma, even after adjustment for confounders (age, family history, breast density) [9]. This suggests that atypia is not merely a marker but is likely part of the causal pathway.

Observational cohorts of ADH (particularly “pure ADH” without concurrent lesions) show meaningful rates of later carcinoma, consistent with a subset of these lesions evolving over many years [21]. Additionally, in analyses of biopsy upgrade rates (concurrent, initially occult carcinoma), ADH diagnoses on core biopsy are upgraded to DCIS or invasive cancer in a variable proportion (10–30%) depending on biopsy technique, lesion features, and institutional practice. This suggests that in many cases, ADH is a “step on the ladder” adjacent to more advanced neoplasia [21].

In conclusion, atypia is a powerful discriminator of risk and a clinically actionable histologic marker of progression potential.

#### 3.2.4. Microenvironmental and Epithelial Constraints: How Atypia May Loosen Barriers

The molecular architecture of atypical lesions (discussed below) suggests a “halfway house”, offering sufficient neoplastic alteration to confer a selective advantage but not yet enough to cross the barrier into invasion. Therefore, while molecular alterations are necessary, they may be insufficient for progression without concomitant loosening of architectural or microenvironmental constraints.

The progression from benign epithelial proliferation to neoplasia also depends on changes within the cellular microenvironment that relax usual growth constraints. Atypical epithelial proliferation may represent a stage at which these regulatory barriers begin to fail, allowing for architectural disorganization and early invasive potential. As epithelial cells acquire atypical features, the structural integrity and functional role of the myoepithelial layer and basement membrane may become compromised. These barriers, normally responsible for maintaining ductal polarity and inhibiting stromal invasion, can appear attenuated, fragmented, or locally absent, diminishing their suppressive influence on epithelial expansion [41].

In parallel, reciprocal communication between the epithelial and stromal compartments is altered. Atypical cells may produce or respond abnormally to growth factors, cytokines, and matrix metalloproteinases (MMPs) that remodel the extracellular matrix, increase matrix stiffness, and recruit fibroblasts and immune cells, collectively creating a pro-tumorigenic milieu [42]. Within these altered microenvironments, the spatial organization of cells and local mechanical or metabolic gradients may further relax normal controls such as contact inhibition and nutrient diffusion, favoring the outgrowth of more resilient subclones [43].

Additionally, atypical epithelial populations often display increased resistance to apoptosis and enhanced survival under oxidative or replicative stress, enabling their persistence in settings where non-atypical cells would undergo programmed cell death [44]. Overall, atypia may therefore represent not only a morphological manifestation of genetic instability but also a biologically active phase in which epithelial and stromal interactions evolve to permit escape from architectural suppression—effectively bridging the transition from hyperplasia to neoplasia. As such, atypia may not only reflect accumulated mutations but also actively mediate escape from architectural suppression—providing a bridge from hyperplasia to neoplasia. Once a lesion surpasses the threshold of DCIS, the transition to invasion shares many overlapping features and constraints, and the presence of atypia earlier can be seen as a prelude.

Among untreated DCIS, 25–60% are believed to progress to invasive carcinoma over years to decades, depending on grade, molecular subtype, and microenvironment [45]. However, not all DCIS progress to invasive disease. Microenvironmental suppression, immune surveillance, and stromal constraints continue to play a role. In that respect, atypia marks the early loosening of barriers, but further events are required to progress to full invasion [46]. Thus, atypia marks the entry point into the neoplastic continuum, with molecular/architectural permissiveness setting the stage for subsequent progression into full in situ and invasive disease [47].

In AH, reciprocal interactions between epithelial and stromal compartments play a pivotal role in fostering a pro-tumorigenic microenvironment. Atypical epithelial cells secrete or aberrantly respond to growth factors, cytokines, and MMPs, which activate stromal fibroblasts into cancer-associated fibroblasts (CAFs) [47]. These CAFs, identifiable by α-SMA expression on immunohistochemistry, remodel the extracellular matrix by increasing collagen deposition and stiffness, thereby disrupting basement membrane integrity and promoting epithelial–mesenchymal transition (EMT)-like changes [48]. This bidirectional crosstalk also recruits immune cells, such as regulatory T cells and macrophages, which suppress anti-tumor immunity and enhance hypoxia-induced angiogenesis via vascular endothelial growth factor (VEGF). Consequently, these alterations relax normal growth constraints, such as contact inhibition, and create metabolic gradients that favor the clonal expansion of resilient subpopulations, bridging the progression from hyperplasia to neoplasia [21,49].

In conclusion, not all atypical lesions are destined to progress. The molecular and morphological heterogeneity within AH means that some lesions may remain indolent, others may regress, and only a subset evolves. The presence of atypia is necessary but not sufficient; additional factors (extent, molecular burden, microenvironment, host context) also influence the trajectory.

Given the importance of atypia, the pressing translational challenge is to differentiate which atypical lesions will progress from those that will remain stable. Biomarkers such as gene-expression risk scores, mutational burden, spatial heterogeneity metrics, and imaging correlates are being explored [49,50].

#### 3.2.5. Specific Molecular Mechanisms from Hyperplasia to Neoplasia

The progression from MDH to breast cancer entails molecular alterations disrupting homeostasis, fostering genomic instability, and enabling transformation, with variations by subtype risk. Low-risk subtypes, like UDH, show minimal, reversible changes without clonal dominance. Genetic changes are rare, including sporadic somatic mutations or copy-number variations such as 1p/8p losses, and are not associated with broad instability [21]. Epigenetic modifications, such as minor promoter hypermethylation of suppressors, fail to drive progression [49]. The tumor microenvironment (TME) maintains homeostasis, with scant stromal remodeling, minimal immune infiltration, and hypoxia-induced angiogenesis, rendering low-risk lesions mere risk markers with a <2-fold RR compared to the general population [21]. High-risk subtypes, such as ADH and ALH, manifest amplified hallmarks bridging benignity and malignancy via low-grade pathways. Drivers include recurrent *PIK3CA* mutations, activating *PI3K-AKT-mTOR* for survival/proliferation. CNVs feature prominent 16q loss and 1q gains, mirroring DCIS and ER+ invasive ductal carcinoma. Epigenetic changes amplify miR-21 (inhibiting apoptosis) and hypermethylation of *APC/TWIST1*, boosting EMT [45]. The TME promotes progression through CAFs secreting hepatocyte growth factor, regulatory T-cell immune suppression, hypoxia-driven VEGF angiogenesis, and MMPs’ extracellular matrix degradation. These explain the 3- to 5-fold RR, facilitating clonal expansion and senescence escape, while enabling *PIK3CA*-targeted therapies [45].

## 4. Genetic Causes and Mechanisms of Mammary Gland Hyperplasia: From Atypia to Malignant Transformation

The likelihood that a given hyperplastic lesion will progress to cancer correlates with the accumulation of specific genetic mutations and epigenetic modifications. Recent research has revealed how these molecular events, from driver gene mutations (e.g., in *PIK3CA*, *BRCA1/2*, *TP53*) to DNA methylation changes and microRNA dysregulation, can cooperate to drive some hyperplasias along the trajectory to malignancy [51,52].

As we have seen, UDH is a proliferation of ordinary ductal epithelium without atypia and is not considered an actual tumor. Generally, it is polyclonal. Molecular analysis shows that UDH lesions typically lack the consistent clonal genetic alterations seen in atypical lesions [48]. Nevertheless, modern sequencing has detected low-frequency activating mutations in pro-growth pathways, even in some UDH cases: PIK3CA mutations or alterations in the *PI3K-AKT-mTOR* pathway [53]. These early mutations may provide a growth advantage but by themselves are insufficient for malignancy, as UDH generally retains normal architecture and growth control.

From a molecular point of view, ADH is a clonal neoplastic proliferation and is considered a genetically advanced precursor lesion [48]. ADH frequently harbors driver mutations commonly seen in luminal-type breast cancers, such as *PIK3CA* mutations (the catalytic subunit of *PI3K*), which are present in a large fraction of ADH lesions [45]. Copy number aberrations characteristic of low-grade carcinomas are also observed; classic examples include gains on 1q and losses on 16q. Loss of heterozygosity on chromosome 16q, which harbors the *CDH1* tumor suppressor gene, has been detected in some ADH cases. 16q loss is a hallmark of the low-grade pathway and is also frequent in concurrent DCIS or invasive cancers of the luminal A subtype. Overall, the genetic profile of ADH often mirrors that of low-grade DCIS, supporting its role as an immediate precursor [48]. Table 1 summarizes key genetic alterations in ADH and other hyperplasias, while Table 2 provides a more detailed view of key genetic drivers from AH to cancer.

Genetically, lobular neoplasia (ADH and ALH) is defined by inactivation of the *CDH1* gene, which encodes E-cadherin. E-cadherin loss is observed in more than 90% of ALH/LCIS, either through mutations in the *CDH1* gene or 16q22 loss of heterozygosity [45,48]. This loss abrogates cell–cell adhesion and is a defining early step in the lobular lineage of breast cancer development [48]. In addition to *CDH1*, lobular neoplastic lesions often harbor mutations in *PI3K* pathway genes similar to those in ADH. For example, *PIK3CA* mutations are found in a substantial subset of ALH/LCIS and invasive lobular carcinomas [48]. Recurrent mutations in the *AKT1* gene (another *PI3K* pathway oncogene) have also been reported in lobular cancers and may be present even at the in situ stage. Remarkably, lobular neoplasia can be multifocal and bilateral; genetically, independent ALH/LCIS foci in the same breast may arise from a field effect of genetically altered but morphologically normal cells distributed through the lobe. The presence of early *BRCA2* or *TBX3* gene mutations in morphologically normal epithelium has been documented in some cases [48]. This field of altered lobules can give rise to clonally related neoplastic lesions along both ductal and lobular pathways (if additional ductal or lobular lineage-specific hits occur) [48].

While genetic mutations lay the foundation for neoplastic transformation, epigenetic modifications add an additional layer of dysregulation in MDH. Epigenetic changes, heritable alterations in gene expression that do not stem from DNA sequence mutations, are now understood to occur early in breast lesion development [49]. These include DNA CpG Island methylation, post-translational histone modifications, and deregulated expression of *non-coding RNAs* such as *microRNAs*. In hyperplastic breast tissue, epigenetic alterations can silence tumor suppressor pathways or activate oncogenic programs, thereby complementing genetic drivers in promoting cell proliferation and abnormal survival. Importantly, epigenetic changes are reversible in principle, and their presence in precursor lesions raises the possibility of early intervention to halt or reverse progression (though therapeutic implications are beyond the scope of this review) [68].

Aberrant DNA methylation is one of the earliest and most consistent epigenetic abnormalities observed during breast carcinogenesis. Even at the stage of hyperplasia, especially in AHs, specific genes acquire promoter hypermethylation, leading to transcriptional silencing. For example, the promoters of tumor suppressor genes such as *RASSF1A* and APC are frequently hypermethylated in pre-invasive breast lesions, including ADH and DCIS. *RASSF1A* is a cell cycle regulator frequently silenced in breast cancer, and studies have detected *RASSF1A* methylation in a significant fraction of AHs and carcinoma in situ. Similarly, *TWIST1*, *HIN1*, *p16INK4a*, and *BRCA1* are among other genes reported to undergo methylation-associated silencing early in the progression sequence [69]. In lobular neoplasia, in addition to global methylation changes, *CDH1* promoter methylation can serve as a second hit in cases lacking a *CDH1* mutation; in fact, *CDH1* promoter hypermethylation is found in a subset of LCIS/ILC, contributing to *E-cadherin* loss [59]. Overall, the presence of DNA methylation changes in a hyperplastic lesion is a red flag for a biologically advanced state.

*MicroRNAs (miRNAs)* are 20–22 nucleotide non-coding *RNAs* that post-transcriptionally regulate sets of target genes. Altered *miRNA* expression profiles are well documented in breast cancers, and emerging evidence indicates that miRNA dysregulation starts at pre-cancerous stages [70]. In fact, certain *miRNA* changes in hyperplasias may signal the development of malignancy and serve as early biomarkers. It was noted that *oncomiRs* such as *miR-21* and *miR-155*, which promote proliferation and inhibit apoptosis, are progressively upregulated from normal epithelium to ADH to DCIS [69]. These *miRNAs* are key regulators of breast tumorigenesis: for instance, *miR-21* suppresses *PTEN* and other tumor suppressors, while *miR-155* modulates *TP53* and *SOCS1*, thereby driving cell growth. On the other hand, some tumor-suppressor *miRNAs* are found to be downregulated in precursors. A striking recent example is *miR-1297*, which is frequently underexpressed in FEA lesions. Scafetta et al. compared *miRNA* profiles of normal breast epithelium, FEA, and DCIS; *miR-1297* was significantly downregulated in FEA and DCIS compared with normal breast epithelium [49]. *MiR-1297* normally targets the oncogenic receptor *Ephrin-A2 (EPHA2)*, so its loss may unleash pro-proliferative signaling. Functional experiments confirmed that restoring *miR-1297* in breast cells inhibited growth, whereas loss of *miR-1297* enhanced proliferation and altered 3D acinar morphology. Thus, *miR-1297* downregulation represents an early event in mammary neoplastic transformation, potentially driving the progression of FEA toward malignancy [49]. This illustrates how even minute non-obligate lesions can harbor significant regulatory changes.

### 4.1. Mechanism of Malignant Transformation

The process by which ADH transitions into carcinoma involves the accumulation of multiple genetic mutations in key pathways governing cellular growth, survival, and apoptosis [71]. These mutations enable hyperplastic cells to evade normal regulatory controls, fostering an escalating potential for invasive behavior. This lesion bridges benign proliferations and DCIS, with features such as cribriform patterns, micropapillae, and uniform nuclear enlargement, all of which are discernible under microscopy [72]. The existing literature indicates that malignant transformation is not obligatory but is often driven by molecular aberrations, which can manifest as synchronous or metachronous carcinomas [25]. For instance, upgrades from ADH to DCIS or IDC occur in 22% to 65% of cases at excisional biopsy, highlighting histopathological progression marked by increased atypia and loss of myoepithelial integrity [73].

Additionally, the hypothetical multistep model of breast carcinogenesis proposes progression from normal epithelium to invasive carcinoma through intraductal hyperplasia (IDH), with and without atypia, including ADH and DCIS, and cytogenetic and molecular-genetic analyses reveal accumulation of genetic alterations [50]. Comparative genomic hybridization (CGH) and fluorescence in situ hybridization (FISH) studies reveal DNA amplification in the chromosomal region *20q13* in early IDH stages, indicating early cytogenetic changes in presumptive precursors [74].

#### 4.1.1. Gene Expression Changes

The onset of atypia in ADH is frequently associated with alterations in gene expression of oncogenes (e.g., *HER2/ERBB2*, *Cyclin D1*) and tumor suppressor genes (e.g., *TP53*, *BRCA1/2*), which histopathologically manifest as proliferative and architectural abnormalities. Transcriptional profiling of matched normal, ADH, and carcinoma samples reveals upregulation of *ERBB2*, *FOXA1*, and *GATA3* in ADH, correlating with estrogen receptor positivity and luminal phenotypes observable on immunohistochemistry (IHC) [75]. These changes are evident in H-E (hematoxylin-eosin) sections as monotonous cell populations with nuclear enlargement and rare mitoses, distinguishing ADH from UDH by uniform spacing and polarization. In synchronous ADH, gene expression patterns cluster with those of low-grade DCIS, exhibiting enriched pathways in membrane transport, fatty acid metabolism, and phenylalanine metabolism, which may contribute to the cribriform architecture and micropapillary formations observed microscopically [76].

Matrix metalloproteinase-1 (MMP-1) emerges as a potential progression biomarker, with elevated expression in synchronous versus pure ADH, aligning with basement membrane discontinuities visible on periodic acid-Schiff (PAS) staining [25]. Furthermore, hub gene analyses identify *RRM2*, *TOP2A*, *PBK*, *MELK*, and *NUSAP1* as progressively upregulated from normal mammary epithelium to ADH, DCIS, and IDC, which is associated with cell cycle deregulation and proliferation. Histopathologically, this correlates with increased Ki-67 positivity in atypical cells, indicating heightened mitotic activity in transitioning lesions [77]. In ADH, including both ductal and lobular variants, gene signatures exhibit shared alterations, such as downregulation of immune-related genes and upregulation of proliferation markers, which are evident in multifocal lesions characterized by expanded ducts and acini on H-E [50]. *ERBB2* overexpression, even without amplification, is noted in ADH, with IHC revealing moderate cytoplasmic and membranous staining in atypical epithelial cells, potentially driving the transition to *HER2*-positive IDC as seen in 7% of subsequent carcinomas [78].

These expression shifts are more pronounced in premenopausal women, in whom higher-grade features, such as punctate necrosis, predict malignant progression, underscoring hormonal influences on histopathological progression [79]. *Cyclin D1* gene amplification and protein overexpression occur in benign breast disease and ADH, with frequencies similar to normal tissue in non-atypical hyperplasias (amplification 15–19%, overexpression 13%) but higher in ADH (amplification 27%, overexpression 57%), approaching DCIS (35% amplification, 50% overexpression) and IDC levels (25% amplification, 64% overexpression). Assessed via differential Polymerase Chain Reaction (PCR) and IHC, these changes precede histologic alterations but increase in atypical lesions [80]. *BigH3* protein expression decreases progressively from benign tissues to DCIS, lobular carcinoma, and IDC, with benign tissue showing a 23-fold increase compared to infiltrating colloid carcinoma, correlating with malignancy in tissue microarray IHC of 192 cases [81].

In vitro hypoxia models show increased Hypoxia-Inducible Factor-1 alpha (*HIF-1*α), Glucose transporter 1 (*GLUT1)*, and Carbonic Anhydrase IX (*CAIX)* expression, with in vivo IHC absent in normal, DH, or ADH but present in DCIS (*GLUT1* 56.8%, *CAIX* 25.0%) and IDC (*GLUT1* 44.1%, *CAIX* 30.5%), higher in high-grade lesions (*p* = 0.001 for GLUT1, *p* = 0.036 for CAIX in DCIS). *Notch1* and *JAG1* hypomethylation inversely correlate with protein overexpression in IDC (*Notch1* 88.7%, *JAG1* 89.9%) versus ADH (36.0%, 45.0%), and are associated with lymph node metastasis and tumor, node, metastasis (TNM) stage [82]. In postmenopausal macaques, *estrogen (E2)* increases proliferation, epithelial area, and progesterone receptor expression (*p* < 0.05), with greater columnar cell hyperplasia in E2-treated groups (*p* < 0.05) and greater *ESR1* and Ki67 in lesional tissue. *MiR-205-5p* downregulation in metastatic 21T series correlates with higher histopathological grades (EG III) and invasion rates [83]. *HER-2* amplification and overexpression are absent in ADH. Still, they are characteristically present in high-grade DCIS, implicating a contributory role in the clonal proliferation underlying neoplastic progression [84].

#### 4.1.2. Loss of Tumor Suppressor Genes

The inactivation of genes such as *PTEN*, *p16*, and *RB* often leads to uncontrolled cell division and contributes to the progression of malignancy, as evidenced by histopathological findings including loss of cellular polarity and increased nuclear pleomorphism. Loss of heterozygosity (LOH) at tumor suppressor loci is an early event in ADH, targeting regions such as 16q (harboring *CDH1/E-cadherin*) and 17p (*TP53*), and is observed in 42% of pure ADH cases via allelic imbalance analysis [85]. This manifests histopathologically as disrupted myoepithelial layers, with reduced *p63* and smooth muscle actin (SMA) expression on IHC, facilitating epithelial-stromal interactions in atypical ducts. In paired UDH and ADH samples, shared *LOH at 11p15.5*, *13q14*, *16q24.3*, and *17p13.1* suggests a continuum, with ADH showing additional losses that correlate with cribriform patterns and partial ductal involvement [86]. *RB1* and *BRCA1/2* regions are implicated, with *16q* deletions being common in low-grade pathways, leading to *E-cadherin* loss and lobular-like features in some ADH lesions, which are visible as expanded acini with uniform cells on H-E [87]. *PTEN* inactivation, though less frequent in pure ADH, accumulates in synchronous cases and is associated with nuclear atypia and mitoses that border on DCIS criteria [88]. Histopathological grading reveals that subsequent carcinomas from ADH exhibit 31% grade 1, 43–53% grade 2, and 16–26% grade 3, with suppressor losses contributing to this spectrum rather than exclusively low-grade disease. *p16* alterations, often epigenetic, are noted in AH, correlating with heterogeneous CK5/6 negativity on IHC, which distinguishes it from UDH’s mosaic pattern and indicates suppressor-mediated dedifferentiation [89].

In high-risk families, ADH prevalence reaches 39%, with *BRCA1/2* mutations enhancing suppressor loss, as seen in prophylactic mastectomy specimens with multifocal atypia and microcalcifications [90]. These inactivations drive morphological shifts, such as the formation of rigid bridges and solid growth, underscoring their role in malignant evolution. Fragile Histidine Triad gene (*FHIT*) and WW domain–containing oxidoreductase (*WWOX*) expressions decrease from normal to ADH, DCIS, and IDC, with higher detectable rates in benign tissue/ADH (rate ratios 2.95–4.58 for *mRNA/protein*) versus in situ/invasive stages [91]. Accumulation of chromosomal imbalances has been documented in premalignant breast lesions, including recurrent gains on chromosomes *3p* and *8q*, as well as losses on *16q*, in ADH, which may implicate tumor suppressor genes in early neoplastic changes [92]. *LOH at 11q13* has been observed in approximately 9% of ADH cases, 0% of low-grade DCIS, and 35% of high-grade DCIS, indicating a potential role for this alteration in facilitating the transition to invasive carcinoma. In intraductal hyperplasia, mutant p53 and cyclin D1 overexpression are more frequently identified in DCIS than in precursor lesions, with LOH at multiple loci associating suppressor gene inactivation with disease progression [93].

#### 4.1.3. Genetic Instability

Atypical lesions tend to harbor genomic instability, including chromosomal aberrations and mutations in critical loci, particularly those involved in DNA repair and cell cycle regulation (e.g., *BRCA1* and *TP53* mutations), which are histopathologically evident as aneuploidy and clonal expansions [94]. Aneuploidy is detected in 15–44% of ADH by flow cytometry and in all cases via Fluorescence In Situ Hybridization (FISH) with multiple probes, higher than in non-atypical lesions but lower than in carcinomas. Comparative Genomic Hybridization (CGH) identifies copy number aberrations such as *16q* loss and *1q* gain in pure ADH, mirroring DCIS/IDC, with histopathological correlates including ductal spaces filled with monotonous cells and pseudo-lumens [95]. Next-generation sequencing in small cohorts reveals shared aneuploidy (e.g., *1q* gain) and somatic mutations, but with intra-lesional heterogeneity in 46% of multifocal ADH, manifesting as mixed clonal proliferations on H-E. *TP53* mutations accumulate in synchronous ADH, associating with nuclear pleomorphism and mitoses, while *BRCA1* defects enhance instability, as seen in high-risk lesions with frequent *LOH* [52].

Centrosomal abnormalities, with increased α- and γ-tubulin expression on IHC, are observed in ADH and carcinoma, indicating mitotic spindle defects that correlate with aneuploidy and invasive foci. Molecular profiles reveal advanced changes, including gross chromosomal rearrangements and epigenetic alterations in AH, with histopathological features such as calcifications and multifocality predicting greater instability and upgrade risk [96]. In progression models, instability drives diploid ADH to aneuploid high-grade breast carcinoma, though most ADH-associated cancers are *ER*-positive and moderate-grade, suggesting a low-grade pathway with occasional escalation. Allelic imbalance frequencies are <5% in benign tissue and ADH, 20% in DCIS, 25% in invasive carcinomas, with significant differences between ADH and DCIS (*p* < 0.0001), suggesting instability predetermines biology at the in situ stage [97].

Genetic alterations are absent in fibroadenomas, even associated with cancer, but *LOH* in DCIS within fibroadenomas, indicating no role in carcinogenesis [98]. *c-erbB-2* amplification is absent in hyperplasia and ADH, but present in high-grade DCIS (10–40% in IDC), highlighting instability in the precursor to invasive transition [99].

#### 4.1.4. Microenvironmental Factors

The surrounding tissue microenvironment, including stromal changes and inflammatory responses, can promote the progression of atypical lesions into invasive cancer, with histopathological evidence of field cancerization and multifocality [100]. Ipsilateral predominance of subsequent breast carcinoma (2:1 ratio) suggests a microenvironmental field effect, persisting post-excision, as observed in cohorts with 80% ipsilateral events in the first 5 years [31].

Stromal alterations, such as desmoplasia and calcification, are common in ADH, as revealed by H-E staining, which shows periductal fibrosis and immune infiltrates that promote progression [101]. Lobular involution extent inversely correlates with risk, with absent involution (SIR 7.66) associating with denser stroma and atypical expansions [102]. Inflammatory responses, including downregulation of immune genes in AH, contribute to evasion, as evidenced histopathologically by reduced lymphocytic aggregates in progressing lesions [103].

Microenvironmental hormones, like in premenopausal women, heighten the risk of higher-grade carcinomas, with features like punctate necrosis on core biopsy predicting upgrade [104]. Field cancerization explains the multifocal appearance of ADH (higher risk if calcified), with non-clonal origins in cancer-prone tissue, as seen in heterogeneous lesions separated by stroma [105]. Tamoxifen’s risk-reduction benefits support modulation of the microenvironment, with histopathological reversal of atypia in treated cases [106].

Overall, these factors, along with genetic changes, drive transformation, advocating for microenvironment-targeted interventions. Extra-tumoral tissue in breast carcinoma patients shows ductal hyperplasia effects similar to non-neoplastic tissue under oral contraceptive use, indicating stromal-inflammatory influences [107]. In progression, hypoxia shifts HIF-1α’s role from a proliferative response in early stages (DH, ADH) to tumor promotion, with GLUT1/CAIX in DCIS/IDC adapting to hypoxia/acidosis to promote aggressive phenotypes [108]. E2 in macaques induces columnar cell changes/hyperplasia, with greater Estrogen Receptor-A1 (ESR1)/Progesterone Receptor (PGR)/Ki67 in lesions, suggesting a hyper response in terminal ductal lobular units [109].

## 5. Epigenetic Modifications in Atypical Hyperplasia and Their Role in Malignant Transformation

### 5.1. General Features

**Figure 2:**
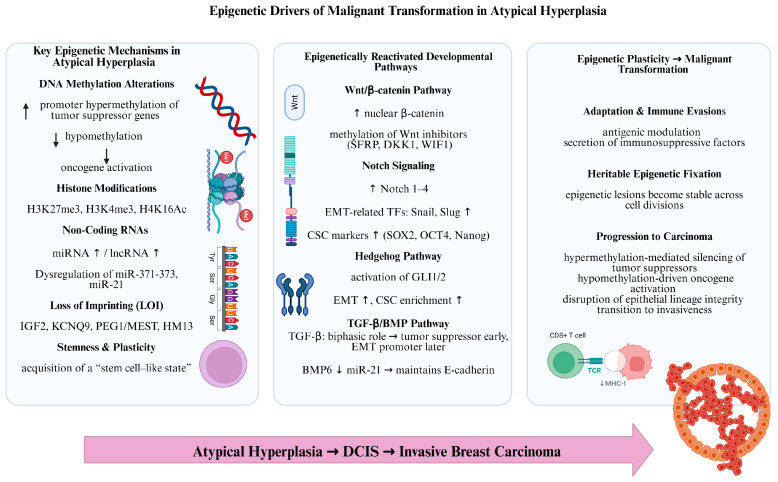
Epigenetic drivers of malignant transformation in atypical hyperplasia (AH) of the breast. Created in BioRender. Elena Mihaela, J. (2025) https://BioRender.com/r65vbpl, accessed on 26 November 2025. Figure legend: DNA—Deoxyribonucleic Acid; *H3K27me3*—Histone 3 lysine 27 trimethylation; *H3K4me3*—Histone 3 lysine 4 trimethylation; *H4K16Ac*—Histone H4 Lysine 16 Acetylation; RNA—Ribonucleic acid; *IGF2*—Insulin-like growth factor 2; *KCNQ9*—Potassium voltage-gated channel subfamily Q member 9; *PEG1/MEST*—Paternally expressed gene 1/Mesoderm-specific transcript; *HM13*—Histocompatibility minor 13; *Wnt*—Wingless Int-1; *SFRP*—Secreted Frizzled-Related Proteins; *DKK1*—Dickkopf-related protein 1; *WIF1*—Wnt Inhibitory Factor 1; *NOTCH*—Neurogenic locus notch homolog protein; EMT—epithelial-to-mesenchymal transition; CSCs—cancer stem cells; *SOX2*—SRY (Sex-determining Region Y)-Box 2; OCT4—Octamer-binding transcription factor 4; TGF-β—Transforming Growth Factor beta; BMP—Bone Morphogenetic Protein.

Epigenetic reprogramming plays a key role in mammary tumorigenesis, favoring the progression from AH to invasive carcinoma by altering gene expression, promoting a stem cell-like state, and enabling EMT [110]. The main epigenetic mechanisms comprise alterations in DNA methylation, histone modifications, non-coding RNA expression, loss of genomic imprinting, and reactivation of developmental pathways such as *Wnt/β-catenin* and *Notch* signaling, which could disrupt tumor suppressor genes and promote oncogenes [111]. Another important epigenetic mechanism is cell plasticity and stemness [112]. Thus, epigenetic reprogramming may confer stem cell-like properties to tumor cells, enabling limitless self-renewal and supporting tumor heterogeneity. Still, these identical flexible epigenetic changes offer therapeutic opportunities, as preventing epigenetic converters may reestablish normal gene expression, making tumors responsive to therapy and improving the efficiency of immunotherapies [113]. Figure 2 offers an overview of the main epigenetic changes in AH.

### 5.2. The Main Epigenetic Mechanisms Involved in Breast Tumorigenesis

#### 5.2.1. Loss of Imprinting

It was found that genomic imprinting has a pivotal role in development and evolution. Loss of imprinting (LOI) represents an initial phase alteration in neoplasia. Imprinting is defined as the monoallelic expression of genes in a parent-of-origin-specific approach [114]. It was shown that in diploid eukaryotes, the maternal and paternal copies of major genes are expressed at equal levels. Concerning imprinted genes, nonetheless, just one allele is transcriptionally dynamic [52]. Imprinting models are variable among tissues [115]. Most imprinted genes are regulated by imprinting control regions, which are generally modulated by DNA methylation, yet *H3K27me3* also contributes to this regulation [116,117]. Moreover, as the number of imprinted genes is fundamental, disruption of imprinting is associated with some human imprinting syndromes and could lead to cancer by promoting oncogenic or suppressing antitumor processes [118,119].

LOI develops in a biallelic expression from the stimulation of the dormant allele. Research in mice revealed that demethylation of imprinted genes leading to LOI made cells more sensitive to cellular transformation and carcinogenesis [52]. Initiation of a growth-promoting allele leads to aberrant cell multiplication, a trigger of cancer [120].

LOI represents an essential abnormality in breast neoplasia growth and evolution, concerning the aberrant expression of imprinted genes linked to epigenetic dysregulation, usually DNA hypomethylation [31]. Reports have discovered imprinted genes such as *HM13*, *KCNQ9*, *IGF2* and *PEG1/MEST* that display LOI in breast cancer, directing to modified gene expression responsible for tumor progression, with possible diagnostic and therapeutic values [31,100].

Research highlighted that *HM13* shows LOI and expression upregulation in mammary tumors, involving DNA demethylation and possibly related to better prognosis in different types of cancer [121].

LOI at the *KCNQ9* locus has been involved in the overexpression of the *TASK3* potassium channel and is linked to triple-negative breast cancer [122]. The connection between *KCNQ9* hypomethylation and triple-negative breast cancers indicates that targeting the *KCNK9* DMR may represent a probable approach for inhibiting this form of breast cancer [102].

Persistent LOI of *PEG1/MEST* has been detected in invasive breast carcinomas, indicating its role in the evolution from hyperplasia to invasive cancer [31]. LOI of the *IGF2* gene could determine its overexpression, supporting the growth of tumor-initiating cells and overall chromatin variability [123].

#### 5.2.2. Reactivation of Developmental Pathways

Several of the best-described signaling pathways influencing self-renewal and differentiation in mature stem cells, like *Wnt/β-catenin*, *Notch*, *Hedgehog* and *TGF-β/BMP* pathways are commonly regulated in cancer via epigenetic processes [91].

Abnormal epigenetic changes in ADH could restart developmental paths, for example *Notch signaling* and *Wnt/β-catenin* signaling, that are usually suppressed in mature cells but are reactivated through cancer development [91,93].

*Notch* signaling controls cell multiplication, differentiation and influences cell outcome and programmed cell death [93]. *Notch* signaling is controlled via *Notch* receptors (1–4) and ligands for instance *Delta-like ligand* (DDL 1/3/4) and Jagged 1/2 [124]. *Notch* signaling collaborates with transcription factors of EMT (*Snail* and *Slug*) and stemness (*SOX2*, *Nanog*, and *OCT4*), in addition to accelerating the acquisition of both EMT and stemness [125]. *Notch*, in combination with *TGF-β*, induces *Slug* expression to modulate EMT. Furthermore, *Notch* signaling controls distinct target genes associated with cancer stem cells (CSCs) and facilitates chemoresistance [126]. For example, *IL-6*, a *Notch* target gene, sustains the self-renewal capacity of CSCs, and *Notch*-regulated activation of *PKB* protects cells from programmed cell death, thereby promoting survival [106]. Inactivation of *Notch* signaling destroys precursor cells that are like CSCs, which promote the function of *Notch* in stemness and correlate with chemoresistance. *MDR1* is highly expressed in CSCs, during which the *Notch* signal is linked to *NF-κB* and related *PI3K/Akt* activation, thereby controlling MRP2, a carrier that facilitates stemness maintenance [91].

*Wnt* signaling pathway, which regulates the expression of target genes via β-catenin, represents a key mechanism of stem cells and CSCs and is abnormally activated through the growth of several human cancers [93]. Acquisition of function mutations of the *CTNNB1* gene (encoding β-catenin) and deficit-of-function mutations of *AXIN* and other genes were observed to be the principal processes responsible for *Wnt* signaling alteration in cancers [127]. Several genes implicated in the *Wnt/β-catenin* signaling pathway are methylated and suppressed in mammary gland cancer, including the *Wnt* inhibitors *SFRP1-5*, *WIF1*, and *DKK1*, as well as the *SRY*-box containing gene 17 (*SOX17*) and *APC* [107]. Current findings indicate that the *Wnt/β-catenin* pathway may also be regulated by histone mutations in cancer [93]. Overexpression of EZH2 in the mammary gland leads to nuclear accumulation of β-catenin and activation of the Wnt pathway, as well as intraductal epithelial hyperplasia [128]. Moreover, the transcriptional suppression of the *Wnt* antagonist *DACT3* was associated with histone modifications at *H3K4me3* and *H3K27me3* [129]. Additionally, *Dkk-1*-mediated suppression of expression was caused by reduced *H4K16Ac* and increased *H3K27me3*, plus the recruitment of *Suz12*, *SirT1*, *EZH2*, and *BMI1* to its promoter [130]. Also, *miRNAs* play a key role in modulating various components of the *Wnt/β-catenin* pathway. It was shown that *β-catenin/Lef1* transactivates the *miR-371-373* group associated with CSCs self-renewal, and, in turn, these *miRNAs* control the *Wnt/β-catenin* signaling by directing *DKK1* expression [131].

*Hedgehog (Hh)* signaling modulates the proliferation of stem and precursor cells in several tissues, and alterations in this pathway have been implicated in tumor growth [93,132]. *Hh* signaling pathway cooperates with *Wnt-β-catenin* signaling, inducing tumorigenesis and cancer severity [133]. This mechanism could regulate both CSC and EMT pathways by increasing key transcription factors (*Slug*, *ZEB1*, *Snail*, *ZEB2*, *FOXC2*, and *TWIST2)* and stem cell markers (*CD44*, *BM1*, and *CD133*) [113]. Enhancement of abnormal *Hh* signaling mechanisms in destructive, metastatic, and chemoresistant mammary gland cancer types leads to reduced *E-cadherin* expression and increased expression of *Twist*, *FOXC2*, *SIP1*, *Snail*, vimentin, *N-cadherin*, and fibronectin [105].

*BMP/TGF-β* signaling pathways control numerous biological processes like proliferation, differentiation, and programmed cell death [91]. Deregulating the molecular effectors of *TGF-β* signaling could lead to cancer [93]. *TGF-β* functions as a tumor suppressor in tumor induction. The initial phases of tumor development and the deactivation of the *TGF-β* tumor suppressor pathway represent the principal phase in the growth of many tumors [134]. Still, in the final phase, it produces tumor progression, EMT, and metastasis [93]. *BMP-6* acts as a suppressor of mammary cancer EMT by saving *E-cadherin* expression. Recent findings suggest that this mechanism is facilitated by *BMP-6*-stimulated transcriptional suppression of *miR-21*, which is overexpressed in destructive mammary gland neoplasia [135].

### 5.3. Epigenetic Plasticity

Epigenetic plasticity enables cells to dynamically alter gene expression patterns in response to the evolving TME, facilitating adaptation and survival under stressful conditions such as hypoxia or nutrient scarcity. This adaptability also allows cancer cells to circumvent intrinsic cellular checkpoints that typically restrict aberrant proliferation, including apoptosis and cell cycle arrest mechanisms [136]. Furthermore, it promotes immune evasion, enabling tumor cells to persist and thrive by modulating surface antigens or secreting immunosuppressive factors. The underlying processes involve key epigenetic modifications, such as histone acetylation or methylation, aberrant DNA methylation at promoter regions, and dysregulation of noncoding *RNAs*, which collectively enhance this phenotypic flexibility during early neoplastic stages [137].

These epigenetic alterations can become heritably fixed through successive cell divisions, stabilizing a malignant phenotype that supports sustained tumor growth. In the context of breast cancer, such changes drive progression from premalignant lesions like ADH by repressing tumor suppressor genes (e.g., via hypermethylation) or activating oncogenes, thereby disrupting lineage integrity and promoting invasive characteristics [138]. Environmental factors, including dietary influences or endocrine disruptors, can further amplify these epigenetic shifts, underscoring the interplay between extrinsic cues and intrinsic molecular reprogramming in cancer evolution [139].

Table 3 highlights some known epigenetic alterations in different hyperplasia subtypes, while Table 4 provides more details on the molecular and cellular effects.

## 6. Tumor Microenvironment Changes in Driving Malignant Transformation of Atypical Hyperplasia

The TME in breast ADH encompasses a multifaceted, evolving landscape comprising neoplastic epithelial cells, stromal fibroblasts, immune cells, vascular networks, and extracellular matrix (ECM) components. These components interact synergistically to modulate the progression from premalignant states, such as ADH, to DCIS and ultimately to IDC. Subtle TME modifications in ADH, such as early stromal fibrosis, immune cell recruitment, and alterations in the basement membrane, are detectable through IHC and serve as harbingers of malignant progression. The existing literature highlights that these TME shifts, including disruption of the myoepithelial layer and activation of fibroblasts, are pivotal in facilitating invasion. For instance, reduced myoepithelial continuity in ADH lesions, as assessed by the co-expression of alpha-smooth muscle actin (α-SMA) and p63 via multiplex IHC, correlates with an increased risk of progression to DCIS. Moreover, gene expression profiling and spatial analyses reveal transcriptional reprogramming in the stroma that supports epithelial invasion. This review synthesizes histopathological evidence from recent studies, highlighting how TME dynamics drive the malignant transformation of ADH, with implications for biomarker development and targeted interventions to halt disease progression.

### 6.1. Key TME Components and Their Impact on Malignant Transformation

#### 6.1.1. Stromal Remodeling

Remodeling in ADH involves the activation and phenotypic shift in fibroblasts, leading to desmoplasia and a supportive niche for epithelial proliferation and invasion. Histopathologically, early ADH exhibits periductal fibrosis with an increased density of α-SMA-positive CAFs, which are observable on IHC as spindle-shaped cells encircling atypical ducts [145]. These CAFs secrete cytokines and growth factors, such as hepatocyte growth factor (HGF), which upregulate the *MET* receptor in epithelial cells, fostering morphological changes indicative of invasive potential. In coculture models mimicking ADH progression, premalignant cells like *MCF10DCIS* display upregulated *HGF/MET* signaling when interacting with mammary fibroblasts, resulting in altered acinar structures in three-dimensional (3D) cultures, characterized by delayed cavitation and increased apoptosis upon HGF blockade [146].

This stromal-epithelial crosstalk is evident in vivo, where high HGF expression in the stromal compartment correlates with basal-like subtypes and poorer survival, highlighting stromal contributions to ADH malignant transformation. Furthermore, myoepithelial cells in ADH undergo phenotypic alterations, as indicated by IHC, which reveals reduced continuity—measured as increased distances between *α-SMA/p63* co-positive cells—compared to normal tissue. This discontinuity, significantly lower in ADH bordering DCIS, permits greater epithelial-stromal interactions, potentially allowing immune cell access and promoting progression [41]. In obesity-associated models, high-fat diet (HFD)-induced stromal changes accelerate ADH formation, with H-E sections showing expanded atypical ducts and DCIS-like lesions; weight loss reverses this by reprogramming the kinome, downregulating *PKC-α* and *MEK3* while upregulating *AMPKα*, as detected by proteomic arrays in unaffected glands [147]. Additionally, inhibitor of differentiation-1 (Id-1) overexpression in ADH, assessed by IHC, correlates with morphologic progression, driving shifts in carcinoembryonic antigen-related cell adhesion molecule 1 (CEACAM1) from apical to cytoplasmic/membranous patterns, disrupting cell adhesion and facilitating stromal invasion [148]. Stromal fibroblasts in high-grade ADH-adjacent areas lose CD34 expression while gaining α-SMA, as shown by IHC, and inversely correlate with microvessel density (MVD), indicating a desmoplastic response that stiffens the matrix and aids epithelial migration [149]. These histopathological features underscore stromal remodeling as a critical driver, and early interventions targeting CAFs may arrest ADH progression.

#### 6.1.2. Immune Cell Infiltration

The immune landscape in ADH’s TME features progressive infiltration of lymphocytes and macrophages, which can either suppress or promote malignant transformation depending on their phenotype and spatial distribution [150]. Histopathologically, H-E staining of ADH biopsies reveals periductal lymphocytic aggregates, with higher stromal lymphocyte counts predicting upgrade to DCIS or IDC. In B3 lesions, including ADH, elevated stromal lymphocytes, quantified per international guidelines, combine with patient age and lesion type to yield a predictive model for upgrade, suggesting immune surveillance failure in progressing cases [151]. Multiplex IHC further delineates this, showing increased tumor-infiltrating lymphocytes (TILs) in *TP53*-mutated ADH, with *CD3+CD8+ T* cells associated with favorable outcomes and CD3+*Foxp3*+ regulatory T cells (Tregs) linked to recurrence. Spatial proximity analyses via IHC demonstrate that closer T cell-tumor cell interactions correlate with reduced invasive risk, implying immune-mediated containment in non-progressing ADH [41].

In hypoxic conditions, myeloid cells release *S100A9*, which fosters systemic immunosuppression, as evidenced by altered CD4+/CD8+ ratios in lymphoid organs and reduced T cell proliferation in cocultures [152]. Histologically, ADH progressing to DCIS shows enhanced stromal immune infiltrates, including macrophages, on H&E, contributing to ECM degradation via protease secretion [153]. In basal-like precursors, immune evasion is marked by increased polymorphonuclear neutrophils (PMNs) in the stroma, as detected by Ly6G+ IHC, which inhibit T cell function and promote atypical hyperplasia [130]. These findings are compounded in obesity, where HFD elevates inflammatory cytokines, leading to denser immune infiltrates in ADH lesions, which are reversible with weight loss, restoring lean phenotypes and reducing pre-neoplastic inflammation. Overall, histopathological evidence points to a shift from anti-tumor to pro-tumor immune profiles in progressing ADH, with TIL density and subtype serving as prognostic indicators [154].

#### 6.1.3. Hypoxia and Angiogenesis

Hypoxic niches in expanding ADH lesions trigger angiogenesis, providing sustenance for proliferation and invasion. Histopathologically, central necrosis in atypical ducts on HE signals hypoxia, with IHC showing nuclear hypoxia-inducible factor-1α (HIF-1α) stabilization in epithelial and stromal cells. This induces vascular endothelial growth factor (VEGF) expression, which correlates with elevated MVD as measured by CD31 IHC, particularly in high-grade ADH transitioning to DCIS [155]. Dynamic contrast-enhanced MRI (DCE-MRI) quantifies this angiogenic switch, revealing increased vessel permeability and a higher vascular fraction in atypical hyperplasia, which persist through progression and are confirmed by microvessel counting [156]. In transgenic models, hypoxia-driven small extracellular vesicles (sEVs) containing HIF-1α promote angiogenesis, as evidenced by CD31 staining showing increased microvessel density and in vivo probes confirming vascular proliferation [133].

Angiogenesis precedes significant stromagenesis in ADH, as evidenced by VEGF-positive epithelial cells and immature vessels on IHC, fostering a permissive environment for invasion.

In obesity contexts, *HFD* exacerbates hypoxic signaling, accelerating angiogenic changes in ADH, while weight loss mitigates this via kinase modulation, reducing vascular support for progression [157]. *HGF/MET* pathways also contribute, with upregulated signaling in ADH, leading to tortuous vessels observable in 3D models. These histopathological markers highlight hypoxia and angiogenesis as early drivers, with therapeutic targeting of *HIF-1α* or *VEGF* potentially preventing ADH malignant evolution.

#### 6.1.4. Extracellular Matrix (ECM) and Cell–Cell Interactions

ECM remodeling in ADH disrupts the structural integrity of the epithelium, enabling epithelial invasion. Histopathologically, Masson’s trichrome staining reveals disorganized collagen in periductal stroma, with IHC showing elevated MMPs from activated fibroblasts degrading basement membranes. Basement membrane discontinuities, visible on PAS staining, coincide with myoepithelial loss, marked by reduced α-SMA/p63 expression, facilitating EMT-like changes with vimentin positivity in atypical cells. *RNA* sequencing of ADH tissues identifies upregulated ECM-related genes, correlating with increased colony formation and migration in Matrigel assays, indicative of invasive potential [158].

Cell–cell adhesions weaken, with IHC demonstrating *E-cadherin* loss and *CEACAM1* redistribution from apical to cytoplasmic patterns in progressing ADH (*p* < 0.05), driven by Id-1 overexpression. In 3D cultures, *HGF/MET* activation induces morphological shifts, such as epithelial budding and ECM invasion, reversible by signaling inhibition [159]. Obesity amplifies ECM stiffening via kinase upregulation, promoting the ADH-to-DCIS transition, while weight loss restores matrix homeostasis. These changes, including enhanced integrin-mediated signaling, underscore ECM and adhesion disruptions as key histopathological features of ADH transformation [160].

#### 6.1.5. Exosomes and Tumor Signaling

Exosomes in ADH’s TME mediate intercellular signaling, transferring oncogenic cargo to remodel the niche. Although direct visualization is challenging, indirect evidence from IHC indicates exosome-driven alterations, such as nuclear accumulation of HIF-1α in recipient cells [129]. Hypoxic sEVs from premalignant cells induce AH, as evidenced by H-E staining, which reveals ductal expansion and increased Ki-67+ proliferation. These vesicles reprogram progenitors, disrupting the polarity of cytokeratin 8/14 and promoting EMT via *RNA*-seq-identified pathways [161]. In progression models, exosomal HIF-1α accelerates oncogene-driven tumorigenesis, and plasma HIF-1α levels correlate with recurrence in luminal cancers. Stromal responses, including *α-SMA* upregulation, suggest exosome-mediated *CAF* activation, though limited histopathological data exists [144]. Targeting exosomal cargo may offer novel strategies to interrupt ADH signaling networks [162].

In conclusion, the histopathological evolution of the TME in ADH—encompassing stromal activation, immune shifts, hypoxic angiogenesis, ECM degradation, and exosomal communication—critically drives malignant transformation. Integrated analyses from IHC, imaging, and molecular profiling provide robust evidence for these mechanisms, advocating for TME-targeted biomarkers and therapies to mitigate progression risks in high-risk patients.

## 7. Diet and Environmental Factors Influencing the Transformation of Atypical Ductal Hyperplasia (ADH) into Breast Cancer

### 7.1. Dietary Factors Influencing ADH Progression

According to the American Cancer Society, about 30% of the breast cancers in postmenopausal patients are related to modifiable risk factors, including diet [163]. Moreover, some research suggests that a healthy diet could prevent breast cancer onset, improve therapeutic outcome and decrease the risk of recurrence among these patients [164].

High-fat diet is an important risk factor for the onset and progression of breast cancer, through multiple pathogenetic mechanisms, involving increased systemic inflammation and oxidative stress [165]. Increased intake of total and saturated animal fat, found in red meat and high-fat dairy, was linked to the risk of breast cancer due to activation of pro-inflammatory mechanisms such as the *NF-kB* signaling, with downstream expression of cytokines, growth factors, and other molecules capable of supporting tumor progression [159,166]. The association between intake of monounsaturated fatty acids and breast cancer appears to depend on the source (olive oil or margarines) and the food processing methods. Intake of olive oil could have a beneficial effect by reducing insulin resistance, whereas hydrogenated oleic acids found in margarines seem to increase breast cancer risk. Polyunsaturated fatty acids can exert different effects on breast cancer, depending on the position of the double bond [167]. Thus, ω-6 fatty acids, such as linoleic acid and arachidonic acid, may promote mammary tumorigenesis by producing eicosanoids and by undergoing oxidation, which results in enhanced cell damage [168]. By contrast, marine ω-3 fatty acids seem to have a protective effect [146].

A high-fat diet can disrupt the gut microbiota, increasing the risk of malignancies, by the activation of the “gut-bone marrow-tumor” axis. Altered microbiota can produce large amounts of leucine, capable of activating the *mTORC1* signaling in myeloid progenitor cells, resulting in differentiation of polymorphonuclear myeloid-derived suppressor cells (*PMN-MDSCs*). An increased number of *PMN-MDSCs* infiltrating the tumor promotes breast cancer growth and metastasis [169].

High-fat diets may promote tumor cell metastasis by preactivating platelets and endothelial cells and by forming premetastatic niches. Within niches, the crosstalk between activated platelets, endothelial cells and cancer cells promotes metastasis [170].

Additionally, high-fat diets are a major driver of obesity, which itself increases breast cancer risk and progression through complex signaling pathways affecting cell growth and angiogenesis [171].

Obesity is a high-risk factor for breast cancer and poor prognosis in women after menopause [172]. Moreover, the risk of triple-negative breast cancer and mortality post diagnosis is significantly increased in both pre- and postmenopausal obese women, compared with normal-weight women [173].

Potential pathogenic mechanisms underlying these associations include: increased estrogen levels, dysregulated insulin and Insulin-like growth factor (IGF) signaling, altered adipokines released by adipose cells, and chronic systemic inflammation indicated by high levels of C-reactive protein [41,174].

In postmenopausal women, the link between obesity and high estrogen levels is more significant, since, after menopause, the adipose tissue is the main source of estrogen. Excess estrogen derived from androgen aromatization in the adipose tissue, associated with obesity-related decrease in sex hormone binding globulin causes a hormonal imbalance, which is linked to the high risk of breast cancer [175,176]. A meta-analysis reported that obese or overweight breast cancer patients develop larger and higher-grade malignancy tumors, more frequent positive lymph nodes and distant metastasis, lower survival rate and worse overall prognostic [177]. This could be explained by the estrogen’s ability to fuel the growth of hyperplastic cells and increase the risk of DNA mutations [178].

Imbalance in adipokines due to increased mass of adipose tissue is also responsible for direct activation of breast cancer cells, contributing to tumor growth and invasion. Low adiponectin levels and high leptin levels were linked to poor prognosis of breast cancer. Adiponectin has a protective role by inhibiting cancer growth [179]. High leptin levels secreted by excess adipose tissue activate multiple mechanisms, including *Jak2/Stat3*, *MAPK*, *PI3K-AKT*, thus increasing tumor cell proliferation and invasion [180].

Chronic systemic inflammation maintains a pro-inflammatory state in adipose tissue, leading to increased secretion of inflammatory cytokines that promote tumor progression and metastasis. In obese individuals, adipose tissue is infiltrated by numerous macrophages, which form crown-like structures surrounding degenerating adipocytes. The presence of such structures in the mammary adipose stroma has been associated with increased levels of blood glucose, insulin, triglycerides, C-reactive protein and IL-6 [181]. Moreover, these patients had a higher risk of developing breast cancer, tumor metastasis and a lower survival rate [182,183]. The mechanisms underlying the poor prognosis in obese patients in association with the accumulation of macrophages and crown-like structures in the mammary gland include: the activation of pathways such as: *NF-κB*, hypoxia-inducible factor 1-alpha (*HIF-1α*), and *PI3K-AKT-mTOR*.

NF-κB, expressed in human breast cancer tumor cells, supports cancer cells’ survival and differentiation into an aggressive phenotype, and is also responsible for the ET resistance in ER+ breast tumors [184]. Expression of *HIF-1α* in adipose tissue macrophages has been detected in patients with insulin resistance and altered glucose metabolism [185].

*PI3K-AKT-mTOR* pathway controls cell growth and survival and has been demonstrated to cross-talk with estrogen receptor (ER) pathway; thus, alterations of *PI3K-AKT-mTOR* pathway are present in ER+ breast cancer [186].

Insulin resistance, which often accompanies obesity, leads to elevated levels of insulin and IGF, known as potent mitogens. Insulin and IGF-related tumorigenesis occurs by activation of oncogenic pathways such as *PI3K/Ras-Raf* in the affected cells, resulting in cell proliferation and ADH progression [187].

According to data published by the World Cancer Research Fund, a higher risk of breast cancer is linked to alcohol consumption, even in low amounts. Pathogenic mechanisms underlying the association between ethanol intake and mammary tumorigenesis include: oxidative stress by accumulation of reactive oxygen species (ROS), systemic inflammation due to increased production of pro-inflammatory cytokines, and insulin resistance [188]. Breast cancer initiation in patients who consume ethanol is the consequence of hormone level alterations, mainly increasing estrogen by activation of aromatase [189]. Thus, alcohol was found to promote the development of ER+ breast tumors, but not ER-cancers. Additionally, alcohol is converted into acetaldehyde, a carcinogenic metabolite that causes DNA damage and induces epigenetic modifications by abnormal DNA methylation, both of which contribute to cancer initiation and progression [165].

Reducing high-fat foods, especially processed meats, fried goods, and high-fat dairy, and replacing them with unsaturated fats, fruits, vegetables, and the correct supplementation with vitamins and minerals is a key lifestyle recommendation for breast cancer prevention and outcome management [142].

Phytoestrogens are plant-derived compounds found in various foods: fruits, vegetables, grains, legumes such as soybeans, flaxseeds, tofu, and lentils [190]. Due to their molecular structure, these compounds perform a wide range of pharmacologic effects, acting as antioxidants, anti-inflammatories, anti-tumoral agents, and can also be effective as endocrine modulators. Upon binding to the estrogen receptors, phytoestrogens can mimic or regulate the function of estrogen in the body [154]. Phenolic phytoestrogens contain two main groups of active pharmacologic substances: flavonoids (including flavone, isoflavone, flavonol phytoestrogens, dihydroflavone phytoestrogens) and non-flavonoid compounds (mainly terpenoids) [191]. Some studies suggest that phytoestrogens might have a protective role against breast cancer by acting as antagonists to estrogen in the breast tissue, potentially reducing the risk of malignancy in estrogen-sensitive cancers [154]. This protective role is explained by the ability of phenolic compounds to bind to the estrogen receptor and to prevent the interaction of endogenous estrogen with the G protein-coupled estrogen receptor (GPER) [192]. Moreover, the hydroxyl group in xenoestrogens plays a role in performing estrogen-like effects [193]. However, the effect of phytoestrogens on the progression of ADH is still debated, as some studies suggest that they may stimulate estrogen receptors in certain contexts, promoting cell proliferation in hormonally driven breast tumors.

Vitamins can also have anti-cancer properties. Studies suggest that vitamin D may inhibit tumor cell proliferation in mammary hyperplastic lesions, whereas vitamins C, E, and carotenoids exert antioxidant properties [194]. Dietary fibers are beneficial for breast cancer prevention by reducing glucose absorption and glycemic levels, and by regulating the balance of gastrointestinal microbiome [195].

### 7.2. Environmental Factors and Toxins Influencing ADH Progression

Although ADH already reflects a significant factor for the malignant transformation, other environmental and toxic factors such as endocrine disruptor chemicals, air pollution, pesticides, radiation exposure, etc., may have an important role in how ADH lesions evolve [196].

#### 7.2.1. Ionizing Radiations

One of the most powerful environmental factors influencing ADH Progression is ionizing radiation, which directly induces double-stranded DNA breaks and also, in mammary epithelial cells, chromosomal instability. Studies show that even low doses of radiation can cause the activation of DNA repair and cell cycle genes (e.g., *BRCA1*). This leads to a proliferative effect in breast tissue [197].

Studies in mice have suggested that radiation exposure causes the activation of genes that are involved in proliferative and metabolic pathways that play a role in carcinogenesis in the mammary glands [198].

#### 7.2.2. Chemical Endocrine Disruptors

Endocrine disruptors are natural or synthetic substances that are able to affect the physiological functions of the endocrine system [199]. These chemicals (EDCs) represent a class of anthropogenic compounds (e.g., bisphenol A, phthalates, and perfluoroalkyl substances) that mimic or antagonize natural hormones. Low-dose exposure to BPA and DDT significantly increases aromatase (*CYP19A1*) biosynthesis and 17β-estradiol production in mammary epithelial cells, leading to sustained activation and proliferation of Erα [200].

Bisphenol A (BPA) is used to produce polycarbonate plastics (e.g., plastic bottles), and recent studies discovered that exposure to BPA, due to the ability to mimic estrogen, can increase the growth of cancer cells in the breast [201].

#### 7.2.3. Air Pollution

Airborne pollutants and particulate matter (PM) contain a multitude of compounds such as polycyclic aromatic hydrocarbons (PAHs) and metals which can stimulate the progression of mammary cancer [202].

Exposure to PM2.5 can lead to oxidative damage to the DNA and can also increase the expression level of oxidation markers. Cellular responses to these factors are activation of transcription factors, release of inflammatory mediators, etc., which can lead to cellular apoptosis. Studies revealed that urban residents have higher PM2.5 exposure and present a higher risk of developing breast cancer [203].

Polycyclic aromatic hydrocarbons (PAHs) are ubiquitous in the environment and are formed in the organic fuel combustion process. Studies have correlated PAH exposure with development of breast cancer [204].

#### 7.2.4. Pesticides

Pesticides such as organophosphates, organochlorines and fungicides have been proven to increase the risk of breast cancer [205]. Organophosphates, a class of chemicals used as pesticides, are proven to act as endocrine disruptors and can increase the risk of mammary tumor development [206].

Dichlorodiphenyltrichloroethane (DDT), a synthetic organochlorine, is found to be in the adipose tissue and studies revealed that higher levels of DDT was found in women with breast cancer compared to women without cancer and a lower DDT level [207].

#### 7.2.5. General Indications

Lifestyle modifications like weight control and limiting environmental toxin exposure vary in effectiveness for reducing progression from atypical ductal hyperplasia (ADH) to breast cancer in specific populations. In postmenopausal women at higher risk from adipose-derived estrogen, weight management via calorie restriction and exercise lowers incidence by 20–50% [208]. A Mediterranean diet, featuring antioxidants from fruits, vegetables, and omega-3s, adds 10–20% to risk mitigation by curbing inflammation and insulin resistance compared with high-fat diets. For ADH/ALH patients under 45, restricting alcohol to under one drink daily effectively cuts estrogen-driven ER+ proliferation, reducing risk by 10–20%. Vulnerable groups exposed to endocrine disruptors (e.g., BPA, pesticides) gain from avoidance like BPA-free items and organics, potentially lessening genomic instability, though evidence is mainly epidemiological. Tailored combinations yield the greatest benefits in postmenopausal obese and younger high-risk individuals [209].

## 8. Conclusions

This review has examined the complex spectrum of MDH and its potential role as a precursor to breast cancer, highlighting why certain subtypes, especially those with atypia, pose a higher risk of becoming malignant. By combining epidemiologic, histopathologic, molecular, and environmental data, we have emphasized the multifactorial nature of progression from benign proliferative lesions to DCIS and invasive cancer. Key findings show that benign breast lesions are categorized into nonproliferative changes (low risk), proliferative hyperplasia without atypia (moderate risk), and proliferative hyperplasia with atypia (high risk). Atypical ductal hyperplasia and ALH serve as crucial transitional states, both morphologically and genetically bridging benignity and malignancy. These atypical forms display early cancer-related changes, such as *PIK3CA* mutations, *BRCA1/2* dysfunction, and epigenetic modifications, including *miR-21* upregulation, that disturb cellular balance and promote genomic instability. The tumor microenvironment further increases this risk through stromal remodeling, immune dysregulation, hypoxia-driven angiogenesis, and ECM breakdown via MMPs. Modifiable factors worsen these processes: obesity and high-fat diets sustain chronic inflammation through adipokines such as leptin, while environmental exposures to ionizing radiation, endocrine disruptors, air pollutants, and pesticides cause DNA damage and hormonal imbalance, primarily affecting high-risk hyperplasia.

Stratified risk assessments enable targeted monitoring: women with atypical hyperplasia might benefit from increased mammographic screening, chemoprevention, or preventive procedures, while women with non-atypical lesions need routine surveillance. Lifestyle changes, such as weight management and reduced toxin exposure, can help prevent progression. Biomarker-based strategies, including molecular testing for *PIK3CA* or *TP53*, could tailor treatment and identify those who may benefit from early intervention. Environmental links, while strong, often rely on epidemiological links rather than direct mechanistic proof. Future research should focus on long-term cohort studies that integrate omics data to understand progression pathways better. Ultimately, this review advocates for a comprehensive approach to MDH management, combining genetic risk, environmental factors, and personalized prevention strategies to lower breast cancer rates.

## Figures and Tables

**Table 1:** Common genetic alterations in subtypes of mammary ductal hyperplasia (MDH).

Lesion Type	Characteristic Genetic Alterations (Mutations and Copy Number Changes)
Usual Ductal Hyperplasia (UDH)	Polyclonal proliferation with minimal or no clonal driver mutations. Occasional activating *PI3K-AKT* pathway mutations (e.g., *PIK3CA* hotspot mutations) have been detected. It lacks consistent chromosomal aberrations; considered a benign hyperplastic response rather than true neoplasia [48].
Atypical Ductal Hyperplasia (ADH)	Clonal neoplastic lesion sharing many alterations with low-grade DCIS. Frequent *PIK3CA* mutations (on the order of 30–40% of cases). Loss of chromosome *16q* (harboring *CDH1* and other genes) is common. Other recurrent mutations in luminal breast cancer genes (e.g., *GATA3*, *MAP3K1*, *CBFB* genes) may be present. Overall, ADH exhibits a luminal A-like genomic profile indicative of early cancerous change [45].
Atypical Lobular Hyperplasia (ALH)	Almost uniformly associated with E-cadherin loss due to *CDH1* gene inactivation. This occurs via *CDH1* gene mutation or *16q* LOH in the majority of cases. Often accompanied by *PIK3CA* gene mutations similar to those in invasive lobular carcinoma. Occasional mutations in the *AKT1* or *PTEN* genes are reported. The genetic pattern overlaps with that of *ER*-positive lobular carcinoma, except for additional alterations required for invasion [54].
Columnar Cell Lesions (CCL) and Flat epithelial atypia (FEA)	High frequency of early oncogenic mutations despite bland histology. *PIK3CA*-activating mutations are present in 50–60% of cases, making this a hallmark of FEA/CCLs. Some cases show modest copy number changes (e.g., gains on 1q; losses on 16q or 17p). Clonal relationship to adjacent ADH/DCIS is evidenced by shared mutations, supporting CCL/FEA as the earliest morphologic precursor in low-grade tumor evolution [55].

Legend Table 1: *PI3K-AKT* = Phosphoinositide 3-kinase—Protein kinase B signaling pathway; PIK3CA = Phosphatidylinositol-4,5-bisphosphate 3-kinase catalytic subunit alpha; *CDH1* = Cadherin-1; GATA3 = DNA binding protein 3; MAP3K1 = Mitogen-Activated Protein Kinase 1; CBFB = Core-binding Factor Subunit Beta; AKT1 = Serine/threonine kinase 1; PTEN = Phosphatase and tensin homolog; ER = Estrogen receptor.

**Table 2:** Key molecular drivers in the malignant progression of atypical mammary hyperplasia.

Gene/Locus	Pathway/Function	Frequent Alterations in ADH/FEA	Pathological Implication	Reference
*PIK3CA*	*PI3K/AKT* signaling (oncogene)	*H1047R*, *E545K*, *E542K* (missense mutations)	Frequent in FEA/ADH and luminal DCIS/IBC; early driver; sustained proliferation; defines low-grade pathway	[56]
*TP53*	Genome integrity/ apoptosis (tumor suppressor)	*R175H*, *R248Q/W*, *R273H/C* (missense mutations)	Subset of atypia/DCIS on progression trajectory; common in high-grade disease; often with *ERBB2* amplification	[50]
*BRCA1*	Homologous recombination DNA repair (tumor suppressor)	*c.68_69delAG (185delAG)*, *c.5266dupC (5382insC*); numerous truncations	High-risk hereditary context; genomic instability in early clones	[57]
*BRCA2*	Homologous recombination DNA repair (tumor suppressor)	Multiple frameshifts/nonsense variants across exons	High-risk hereditary context; facilitates mutation accumulation	[58]
*GATA3*	Luminal Lineage Transcription Factor	Frameshift/truncations; splice-site alterations	Supports luminal differentiation program in HR+ precursors	[59]
*CDH1*	*E-cadherin*; cell–cell adhesion (tumor suppressor)	Bi-allelic inactivation/truncating mutations	Marks the lobular pathway (atypical lobular hyperplasia to lobular carcinoma)	[60]
*AKT1*	Serine/threonine kinase downstream of *PI3K* (oncogene)	*E17K* (PH domain hotspot)	Activates *AKT* independent of *PI3K*, specific to HR+ lesions	[61]
*PTEN*	*PI3K* negative regulator; lipid phosphatase (tumor suppressor)	Loss-of-function mutations/deletions; promoter silencing	Cooperates with *PIK3CA* activation in atypia/DCIS	[48]
*ERBB2 (HER2)*	Receptor tyrosine kinase (oncogene)	Amplification; *S310F/Y; Y772_A775dup* (kinase insertion)	Low-level expression in some early lesions; targetable alterations	[62]
*ERBB3 (HER3)*	Receptor tyrosine kinase; dimerizes with HER2	*E928G* and kinase-domain substitutions	Co-occurs with *ERBB2* mutations; luminal contexts	[59]
*RUNX1/CBFB*	Core-binding factor complex; lineage and differentiation	*RUNX1* Runt domain loss-of-function; *CBFB* alterations	Luminal tumors/precursors; tumor-suppressor roles	[48]
*MAP3K1*	*MAPK* signaling; luminal A-associated	Multiple truncations/missense; pathway-inactivating	Co-mutation patterns with *PIK3CA/CBFB* in luminal A	[55]
*ESR1*	*ER-alpha* (nuclear receptor)	Ligand-binding domain: *Y537S*, *D538G* (rare in primary)	Rare in early lesions; prevalent in endocrine-resistant metastases	[63]
*FOXA1*	Pioneer transcription factor for the *ER* program	*Wing2/DBD* hotspots (e.g., R219); indels affecting DNA binding	Luminal program maintenance in HR+ tumors; early presence reported	[64]
*KMT2C (MLL3)/KMT2D (MLL2)*	Histone *H3K4* methyltransferases (chromatin modifier)	Truncating mutations across coding exons	Epigenetic deregulation in luminal tumors/precursors	[65]
*ARID1A/ARID1B*	SWI/SNF chromatin remodeling complex (tumor suppressor)	Frameshift/nonsense mutations; loss of protein expression	Subset of HR+/basal lesions; chromatin accessibility shifts	[66]
*NCOR1/NCOR2*	Nuclear receptor co-repressors; *ER* signaling modulation	Truncations and splice variants	Associated with endocrine response and luminal programs	[67]
*CREBBP/EP300*	Histone acetyltransferases; transcriptional co-activators	Loss-of-function mutations	Transcriptional reprogramming in early neoplasia	[65]
*PIK3R1*	*PI3K* regulatory subunit p85α (tumor suppressor-like)	Truncations/indels disrupting SH2 domains	Cooperates with *PIK3CA* in *PI3K* activation	[51]
*NOTCH1*	*NOTCH* signaling (context-dependent)	PEST-domain truncations; activating mutations	Subset of breast lesions; cross-talk with *ER* and *PI3K*	[50]

Legend Table 2: ADH = Atypical Ductal Hyperplasia; FEA = Flat Epithelial Atypia; CIS = Ductal Carcinoma In Situ; IBC = Invasive Breast Carcinoma; HR+ = Hormone Receptor Positive; ER = Estrogen Receptor; *PIK3CA* = Phosphatidylinositol-4,5-bisphosphate 3-kinase catalytic subunit alpha; *TP53* = Tumor Protein p53; *BRCA1* = Breast Cancer Gene 1; *BRCA2* = Breast Cancer Gene 2; *GATA3* = DNA Binding Protein 3; *CDH1* = Cadherin 1; *AKT1* = Serine/Threonine Kinase 1; *PTEN* = Phosphatase and Tensin Homolog; *ERBB2* = Erb-B2 Receptor Tyrosine Kinase 2/HER-2 = Human Epidermal Growth Factor Receptor 2; *ERBB3* = Erb-B2 Receptor Tyrosine Kinase 3/HER3 = Human Epidermal Growth Factor Receptor 3; *RUNX1* = Runt-Related Transcription Factor 1; *CBFB* = Core-Binding Factor Subunit Beta; *MAP3K1* = Mitogen-Activated Protein Kinase 1; *ESR1* = Estrogen Receptor 1; *FOXA1* = Forkhead Box A1; *KMT2C*/MLL3 = Lysine Methyltransferase 2C/Mixed-Lineage Leukemia 3; *KMT2D/MLL2* = Lysine Methyltransferase 2D/Mixed-Lineage Leukemia 2; *ARID1A* = AT-Rich Interaction Domain 1A; *ARID1B* = AT-Rich Interaction Domain 1B; *NCOR1* = Nuclear Receptor Corepressor 1; *NCOR2* = Nuclear Receptor Corepressor 2; *CREBBP* = CREB Binding Protein; EP300 = E1A Binding Protein p300; *PIK3R1* = Phosphatidylinositol-3-Kinase Regulatory Subunit 1; *NOTCH1* = Notch Receptor 1; *MAPK* = Mitogen-Activated Protein Kinase; *SWI/SNF* = Switch/Sucrose Non-Fermentable; SH2 = Src Homology 2; *PEST* = Proline–Glutamate–Serine–Threonine; *DBD* = DNA-Binding Domain; *PH* = Pleckstrin Homology; *PI3K* = Phosphoinositide 3-Kinase.

**Table 3:** Notable epigenetic changes in mammary ductal hyperplasia (MDH) subtypes.

Lesion Type/Stage	Epigenetic Alterations
Early proliferative lesions (UDH, mild hyperplasia)	Subtle epigenetic deviations may begin. Global DNA methylation levels start to increase in proliferative breast tissue. Promoter methylation is generally low in UDH, but isolated foci can show initial methylation of genes like *RASSF1A*. Histones in UDH largely retain normal patterns, although minor shifts toward a more closed chromatin conformation have been noted in high-risk patients. MiRNA expression is near-normal, aside from slight upregulation of proliferative miRNAs (e.g., miR-21) in some cases. Overall, UDH lacks the pronounced epigenetic silencing seen in atypia [140].
Atypical hyperplasias (ADH, ALH, FEA)	Promoter hypermethylation becomes common and non-random. Frequent methylation of tumor suppressor genes (e.g., RASSF1A, APC) is documented in ADH and FEA lesions. In lobular neoplasia, *CDH1* promoter methylation is an alternative mechanism to gene mutation for turning off *E-cadherin* [59]. These methylation marks often coexist with repressive histone modifications; for instance, hypermethylated genes show enriched H3K27me3 and decreased acetylation, reinforcing chromatin compaction. Genome-wide, atypical lesions exhibit a shift toward a cancer-like epigenome with dozens of genes epigenetically silenced. *MicroRNA* profiles are significantly altered: *oncomiRs* such as *miR-21* and *miR-155* are elevated in ADH, correlating with suppressed *PTEN* and *TP53* pathways [141]. Conversely, certain anti-proliferative *miRNAs* (e.g., *miR-1297*, *miR-125b*) are downregulated in FEA/ADH [59]. These changes act in concert to disable cell-cycle checkpoints and promote survival, thereby potentiating progression to carcinoma in situ.
Carcinoma in situ (DCIS, LCIS)	Widespread epigenetic reprogramming is present. DCIS lesions show hypermethylation at numerous loci, e.g., more than 40% of DCIS cases have *RASSF1A* gene methylation [68], and many harbor methylation of *p16*, *cyclin D2*, and others. LCIS (especially pleomorphic LCIS) likewise accumulates methylation events, though classical LCIS may rely more on *CDH1* loss by mutation. Global DNA hypomethylation becomes evident in carcinoma in situ, contributing to genomic instability. Histone modification patterns are clearly abnormal: high levels of HDACs lead to histone hypoacetylation, and Polycomb complexes maintain silencing of differentiation genes. Such miRNA dysregulation in CIS has been linked with aggressive features and may predict which in situ lesions are likely to invade [69].

Legend Table 3: UDH = Usual Ductal Hyperplasia; AHs = Atypical Hyperplasias; ADH = Atypical Ductal Hyperplasia; ALH = Atypical Lobular Hyperplasia; Flat Epithelial Atypia; DCIS = Ductal Carcinoma In Situ; LCIS = Lobular Carcinoma In Situ; LN = Lobular Neoplasia; CIS = Carcinoma In Situ; *RASSF1A* = Ras Association Domain Family Member 1; *APC* = Adenomatous Polyposis Coli; *CDH1* = Cadherin 1; *PTEN* = Phosphatase and Tensin Homolog; *TP53* = Tumor Protein p53;DNA = Deoxyribonucleic Acid; *H3K27me3* = Trimethylation of Lysine 27 on Histone H3; *HDACs* = Histone Deacetylases; miRNA = MicroRNA; miR-21 = MicroRNA 21; miR-155 = MicroRNA 155; miR-1297 = MicroRNA 1297; *miR-125b* = MicroRNA 125b; *miR-210* = MicroRNA 210; *miR-205* = MicroRNA 205; EMT = Epithelial–Mesenchymal Transition. OncomiRs = Oncogenic MicroRNAs.

**Table 4:** Molecular and cellular effects of epigenetic mechanisms driving malignancy in atypical hyperplasia.

Mechanism	Gene Targets/Factor	Molecular Results in ADH/DCIS	Functional Consequences
Promoter hypermethylation (DNA)	*RASSF1*, *CDKN2A* (p16INK4a), *BRCA1*, *PTEN*	Stable silencing of tumor suppressors	Loss of cell cycle arrest; early event in monoclonal progression [142]
Oncogene hypomethylation (DNA)	*HER2*, *CCND1* (Cyclin D1)	Transcriptional increase	Overexpression of proliferation drivers [143]
Non-coding RNA dysregulation	*miR-21* (Upregulation)	Post-transcriptional inhibition of *PTEN*	Constitutive activation of the *PI3K/AKT* pathway; promotes survival [144]
Histone modifications/remodeling	*KMT2C/D*, *EZH2*	Aberrant acetylation/methylation patterns	Transcriptional reprogramming for malignant phenotype [47]

Legend Table 4: ADH = Atypical Ductal Hyperplasia; DCIS = Ductal Carcinoma In Situ; DNA = Deoxyri-bonucleic Acid; *RASSF1* = Ras Association Domain Family Member 1; *CDKN2A* = Cyclin-Dependent Ki-nase Inhibitor 2A; *p16INK4a* = p16 Inhibitor of Cyclin-Dependent Kinase 4a; *BRCA1* = Breast Cancer Gene 1; *PTEN* = Phosphatase and Tensin Homolog; *HER2* = Human Epidermal Growth Factor Receptor 2; *CCND1* = Cyclin D1; *miR-21* = MicroRNA 21; *PI3K* = Phosphoinositide 3-Kinase; *AKT* = AKT Ser-ine/Threonine Kinase; *KMT2C* = Lysine Methyltransferase 2C; *KMT2D* = Lysine Methyltransferase 2D; *EZH2* = Enhancer of Zeste Homolog 2.

## Data Availability

No new data were created or analyzed in this study. Data sharing is not applicable to this article.

## Abbreviations

The following abbreviations are used in this manuscript:

MDH	Mammary ductal hyperplasia
DCIS	ductal carcinoma in situ
RR	relative risk
UDH	usual ductal hyperplasia
OR	odds ratio
ADH	atypical ductal hyperplasia
ALH	atypical lobular hyperplasia
FEA	flat epithelial atypia
MRI	Magnetic Resonance Imaging
LCIS	lobular in situ carcinoma
PIK3CA	Phosphatidylinositol-4,5-bisphosphate 3-kinase catalytic subunit alpha
BRCA1/2	Breast CAncer gene 1/2
TP53	Tumor Protein 53
DNA	Deoxyribonucleic Acid
K-AKT-mTOR	Phosphoinositide 3-kinase-AKR mouse thymoma-Mechanistic (mammalian) Target of Rapamycin
CDH1	Cadherin-1
TBX3	T-box transcription factor 3
CCLs	Columnar cell lesions
ER	Estrogen Receptor
NF1	Neurofibromin 1
GATA	Glutamyl aminotransferase subunit A
MAP3K1	Mitogen-activated protein kinase kinase kinase 1
CBFB	Core-Binding Factor, Beta Subunit
16q LOH	16 q Loss of Heterozygosity
PTEN	phosphatase and tensin homolog
IBC	invasive breast cancers
ERBB2	v-erb-b2 avian erythroblastic leukemia viral oncogene homolog 2
HR	hormone receptors
RUNX1	Runt-related transcription factor 1
FOXA1	Forkhead box protein A1
KMT2C (MLL3)/KMT2D (MLL2)	type 2 lysine methyltransferases
ARID 1A/ARID 1B	AT-Rich Interaction Domain 1A Gene/AT-Rich Interaction Domain 1B Gene
NCOR1/NCOR2	Nuclear Receptor Co-Repressor 1/Nuclear Receptor Co-Repressor 2
CREBBP/EP300	CAMP Response Element-Binding-Binding Protein/E1A-binding protein P300
NOTCH1	Neurogenic locus notch homolog protein 1
RNAs	Ribonucleic acid
RASSF1A	Ras Association Domain Family 1, isoform A
APC	Adenomatous Polyposis Coli
TWIST1	Twist Family BHLH Transcription Factor 1
HIN1	Hypermethylated in Cancer 1
p16INK4a	Cyclin-Dependent Kinase Inhibitor 2A
TP53	Tumor Protein p53
SOCS1	Suppressor of Cytokine Signaling 1
CGH	Comparative genomic hybridization
FISH	Fluorescence in situ hybridization
HER 2	Human Epidermal Growth Factor Receptor 2
IHC	Immunohistochemistry
MMP-1	Matrix metalloproteinase-1
PAS	periodic acid-Schiff
RRM2	Ribonucleotide Reductase Regulatory Subunit M2
TOP2A	Topoisomerase II Alpha
PBK	PDZ-Binding Kinase
MELK	Maternal Embryonic Leucine Zipper Kinase
NUSAP1	Nucleolar and Spindle-Associated Protein 1
AH	atypical hyperplasia
H-E	haematoxylin and eosin
PCR	polymerase chain reaction
HIF-1alpha	Hypoxia-Inducible Factor 1 alpha
GLUT1	Glucose Transporter 1
CAIX	Carbonic Anhydrase IX
JAG1	Jagged 1
RB	Retinoblastoma protein
LOH	Loss of heterozygosity
TNM	Tumor–Node–Metastasis staging system
SMA	and smooth muscle actin
CK5/6	Cytokeratin 5/6
FHIT	Fragile Histidine Triad
WWOX	WW Domain–Containing Oxidoreductase
CGH	Comparative Genomic Hybridization.
c-erbB-2	HER2
SIR	Signal-to-Intensity Ratio
ESR1	Estrogen receptor 1
PGR	Progesterone receptor
Ki-67	Tumor proliferation
EMT	epithelial-to-mesenchymal transition
Wnt	Wingless Int-1
LOI	Loss of imprinting
H3K27me3	Histone 3 lysine 27 trimethylation
HM13	Histocompatibility minor 13
KCNQ9	Potassium voltage-gated channel subfamily Q member 9
IGF2	Insulin-like growth factor 2
PEG1/MEST	Paternally expressed gene 1/Mesoderm-specific transcript
TASK3	TWIK-related acid-sensitive potassium channel 3
KCNK9 DMR	Potassium channel subfamily K member 9 differentially methylated region
TGF-β	Transforming Growth Factor beta
BMP	Bone Morphogenetic Protein
DDL	Delta-like ligand (DLL)
SOX2	SRY (Sex-determining Region Y)-Box 2
OCT4	Octamer-binding transcription factor 4
CSCs	cancer stem cells
IL-6	Interleukin 6
PKB	Protein Kinase B (also known as AKT)
NF-κB	Nuclear Factor kappa-light-chain-enhancer of activated B cells
MRP2	Multidrug Resistance-associated Protein 2
CTNNB1	Catenin Beta 1 (β-catenin)
AXIN	Axis Inhibition Protein
SFRP1-5	Secreted Frizzled-Related Proteins 1–5
WIF1	Wnt Inhibitory Factor 1
DKK1	Dickkopf-related protein 1
SRY	Sex-determining Region Y
EZH2	Enhancer of Zeste Homolog 2
DACT3	Disheveled-binding Antagonist of β-catenin 3
H3K4me3	Histone 3 lysine 4 trimethylation
Suz12	Suppressor of Zeste 12
SirT1	Sirtuin 1
BMI1	B lymphoma Mo-MLV insertion region 1 homolog
Lef1	Lymphoid Enhancer-binding Factor 1
Slug	Snail family transcriptional repressor 2
ZEB1	Zinc Finger E-box-binding Homeobox 1
Snail	Snail family transcriptional repressor 1
ZEB2	Zinc Finger E-box-binding Homeobox 2
FOXC2	Forkhead Box C2
TWIST2	Twist family bHLH transcription factor 2
CD44	Cluster of Differentiation 44
CD133	Cluster of Differentiation 133
SIP1	Smad Interacting Protein 1
BMP-6	Bone Morphogenetic Protein 6
ECM	extracellular matrix
TME	tumor microenvironment
α-SMA	alpha-smooth muscle actin
CAFs	cancer-associated fibroblasts
HGF	hepatocyte growth factor
MET receptor	Mesenchymal–Epithelial Transition receptor
MCF10	original non-tumorigenic human mammary epithelial cell line
HGF/MET	Hepatocyte Growth Factor/MET receptor signaling pathway
HFD	high-fat diet
CEACAM1	in carcinoembryonic antigen-related cell adhesion molecule 1
MVD	Microvessel Density
PKC-α	Protein Kinase C alpha
MEK3	Mitogen-activated protein kinase kinase 3
AMPKα	AMP-activated protein kinase alpha subunit
TILs	tumor-infiltrating lymphocytes
Foxp3	Forkhead box P3
Tregs	regulatory T cells
S100A9	S100 calcium-binding protein A9
PMN	polymorphonuclear neutrophils
Ly6G+	Lymphocyte antigen 6 complex, locus G positive
HIF 1α	1hypoxia-inducible factor-1α
VEGF	vascular endothelial growth factor
DCE-MRI	Dynamic contrast-enhanced MRI
sEVs	small extracellular vesicles
PMN-MDSCs	polymorphonuclear myeloid-derived suppressor cells
IGF	Insulin-like growth factor
MAPK	Mitogen-Activated Protein Kinase
ET	Endocrine Therapy
ROS	reactive oxygen species
GPER	G protein-coupled estrogen receptor
EDCs	Endocrine-disrupting chemicals
BPA	Bisphenol A
DDT	Dichlorodiphenyltrichloroethane
CYP19A1	Cytochrome P450 Family 19 Subfamily A Member 1
PM	particulate matter
PAHs	polycyclic aromatic hydrocarbons
